# Artificial Metalloenzymes: From Selective Chemical Transformations to Biochemical Applications

**DOI:** 10.3390/molecules25132989

**Published:** 2020-06-30

**Authors:** Tomoki Himiyama, Yasunori Okamoto

**Affiliations:** 1 National Institute of Advanced Industrial Science and Technology, Ikeda, Osaka 563-8577, Japan; t-himiyama@aist.go.jp; 2 DBT-AIST International Laboratory for Advanced Biomedicine (DAILAB), Ikeda, Osaka 563-8577, Japan; 3 Frontier Research Institute for Interdisciplinary Sciences, Tohoku University, 6-3 Aramaki aza Aoba, Aoba-ku, Sendai 980-8578, Japan

**Keywords:** artificial metalloenzyme, organometallic catalysis, protein engineering, selective chemical transformation, chemoenzymatic cascade, directed evolution, intracellular catalysis

## Abstract

Artificial metalloenzymes (ArMs) comprise a synthetic metal complex in a protein scaffold. ArMs display performances combining those of both homogeneous catalysts and biocatalysts. Specifically, ArMs selectively catalyze non-natural reactions and reactions inspired by nature in water under mild conditions. In the past few years, the construction of ArMs that possess a genetically incorporated unnatural amino acid and the directed evolution of ArMs have become of great interest in the field. Additionally, biochemical applications of ArMs have steadily increased, owing to the fact that compartmentalization within a protein scaffold allows the synthetic metal complex to remain functional in a sea of inactivating biomolecules. In this review, we present updates on: (1) the newly reported ArMs, according to their type of reaction, and (2) the unique biochemical applications of ArMs, including chemoenzymatic cascades and intracellular/in vivo catalysis. We believe that ArMs have great potential as catalysts for organic synthesis and as chemical biology tools for pharmaceutical applications.

## 1. Introduction

A catalyst is defined as a material that accelerates the rate of a chemical reaction without being consumed by the reaction. To realize unprecedented chemical transformations, chemists have developed various catalysts. In view of their tremendous impact on society in terms of the production of industrial chemicals and pharmaceuticals several Nobel prizes have been awarded for the development of catalysts for the Haber–Bosch process, Ziegler–Natta polymerization, asymmetric syntheses, metathesis, and cross-coupling. As catalysts provide efficient access to a target compound with less energy and fewer steps, they play a major role in sustainable green chemistry.

There are three main types of catalysts, namely, homogeneous catalysts, heterogeneous catalysts, and biocatalysts (enzymes). Biocatalysts allow highly selective reactions under mild conditions, often in water and at ambient temperature and atmospheric pressure. Consequently, biotransformations using purified enzymes and microorganisms expressing enzymes have attracted considerable attention as valuable tools in synthetic chemistry [1,2]. Previously, the shortcomings of biotransformations, such as the narrow substrate scope and limited repertoire of reactions, have been discussed. However, the directed evolution methodology of enzymes, for which the Nobel Prize in Chemistry was awarded in 2018, and artificial metalloenzyme (ArM) technology have shown potential in overcoming these challenges [3]. The reader is referred to several excellent reviews on the directed evolution of natural enzymes [4,5,6]. An ArM comprises a synthetic metal complex in a protein scaffold. A comprehensive review of ArMs, covering examples from the very early stages to 2016, has been published, together with a searchable database [7]. Although only a few years have passed since the publication of that review, dozens of original reports and reviews on ArMs have been published since [8,9,10,11,12,13,14,15,16,17,18,19,20,21,22].

There are four main methods for constructing ArMs, namely, dative anchoring (non-native metal ions or metal complexes are coordinated by the amino acid residues on the protein surface), cofactor replacement (e.g., an iron porphyrin complex, which is the cofactor of hemoproteins, is replaced with a rigid and planar metal complex), covalent anchoring (a metal complex bearing a functional group, such as maleimide, forms a covalent bond with a cysteine residue in post-functionalization of a (semi)isolated protein), and supramolecular anchoring (utilizing supramolecular recognition, such as the biotin–avidin interaction, a high-affinity molecule attached to a metal complex binds to the corresponding protein). Recently, a genetic code reprogramming technique has enabled the incorporation of unnatural amino acids, which can be a ligand for a metal complex or a catalytically active small molecule, into a protein upon its expression. This genetic incorporation of unnatural amino acids is considered as a fifth method for ArM construction [23].

ArMs display performances that merge combine those of both homogeneous catalysts and biocatalysts. Specifically, ArMs facilitate a wide range of non-natural reactions, which are inaccessible to natural enzymes, in water and under mild conditions. High reaction selectivity can also be achieved by ArMs because the protein scaffold provides a highly defined second coordination sphere for the metal complex, which is difficult to implement through synthetic chemistry. Since the early 2000s, when developments in structural biology allowed chemists to consider proteins as designable macromolecules and genetic engineering methods became more accessible to chemists, ArM research has progressed rapidly. An attractive new feature of ArMs is their biocompatibility, which has encouraged further applications [24]. In this review, we first present updates on the newly reported ArMs according to the type of reaction they catalyze and then discuss the unique applications of ArMs that have been reported in the past few years.

## 2. Recent Progress on the Various Reactions Catalyzed by ArMs

### 2.1. Reduction

**Figure 1:**
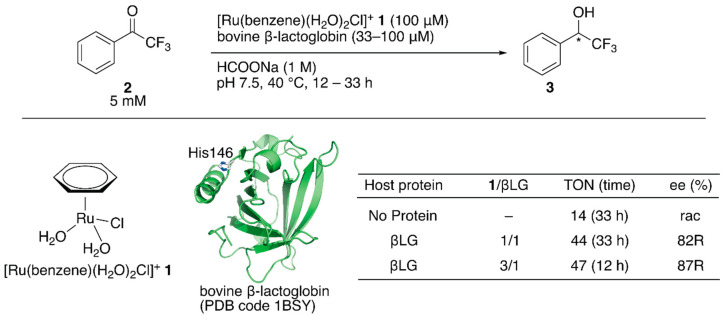
Transfer hydrogenation of ketones catalyzed by **1**·βLG with formate salt as a hydride source. Adapted from [31].

**Figure 2:**
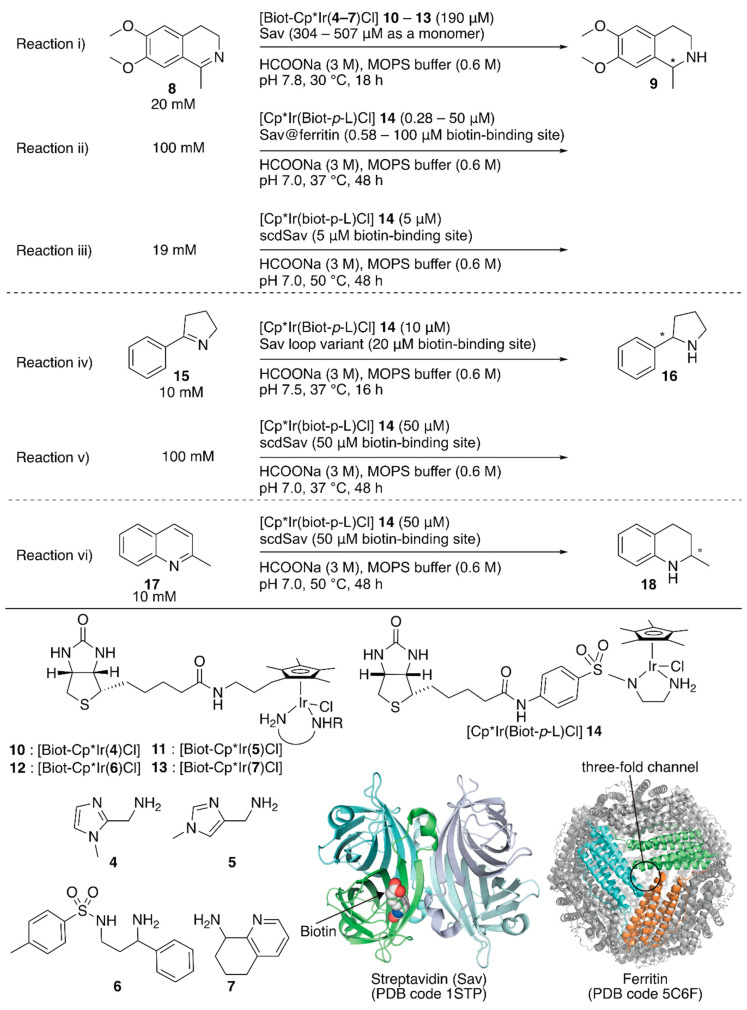
Transfer hydrogenation of imines catalyzed by Sav-based artificial transfer hydrogenases. Adapted from [35,36,37,38].

Chiral alcohols and imines are important intermediates in the production of pharmaceuticals and agrochemicals. Accordingly, the catalytic asymmetric reduction of ketones and imines has received considerable attention. In 1995, Noyori and coworkers used the enantiopure TsDPEN-Ru(II)-based complex (TsDPEN = *N*-(*p*-toluenesulfonyl)-1,2-diphenylethylenediamine) for asymmetric transfer hydrogenation [25]. In contrast to hydrogenation with hazardous H_2_ gas, transfer hydrogenation relies on formic acid or isopropanol as hydrogen donors. To date, various *d*_6_-pianostool organometallic complexes, such as [Ru(benzene)(H_2_O)_2_Cl]^+^ **1** (Figure 1) and [Cp*Ir(Biot-*p*-L)Cl]^+^ **14** (Cp* = pentamethylcyclopentadienyl, Biot = biotin) (Figure 2), have been developed as catalysts for this reaction [26]. Instead of the enantiopure ligand, it has been demonstrated that an achiral complex incorporated within a protein scaffold also displays enantioselectivity. Various *d*_6_-pianostool complexes and protein scaffolds have been combined via the different types of anchoring method mentioned above [7]. Although several excellent ArMs are known to reduce H^+^ [27,28], CO_2_ [29], and SO_3_^−^ [30], we have focused herein on ArMs used as catalysts for the reduction of organic molecules.

#### 2.1.1. Transfer Hydrogenation

Salmain and coworkers modified bovine β-lactoglobulin (βLG) with [Ru(benzene)(H_2_O)_2_Cl]^+^ **1** through dative anchoring to obtain an artificial transfer hydrogenase (Figure 1) [31]. Capping the solvent-accessible His146 of βLG with dimethyl pyrocarbonate resulted in a significant decrease in the catalytic activity and selectivity, suggesting that [Ru(benzene)(H_2_O)_2_Cl]^+^ **1** was directly coordinated to His146. In the transfer hydrogenation of ketone **2**, the bare catalyst **1** yielded racemic alcohol product **3** with a turnover number (TON) of 14, whereas [Ru(benzene)(H_2_O)_2_Cl]^+^ **1**·βLG gave a TON of 44 with 82% (*R*) ee (Figure 1). The catalytic activity of this ArM was further improved by changing the mixing ratio of **1** and βLG. At a ratio of **1**:βLG = 3:1, the turnover frequency (TOF) was 2.3-fold higher than that at a ratio of 1:1, indicating that there were other binding sites of [Ru(benzene)(H_2_O)_2_Cl]^+^ **1** in βLG (Figure 1). However, it was difficult to identify the putative amino acid residues because of the labile coordination of [Ru(benzene)(H_2_O)_2_Cl]^+^ **1**.

Ward and coworkers have demonstrated asymmetric transfer hydrogenation by using streptavidin (Sav)-based ArMs. Sav is a homotetrameric β-barrel protein with an approximate molecular weight of 65 kDa from *Streptomyces avidinii*. Owing to the strong supramolecular recognition between Sav and biotin, the desired metal complex can be accommodated in the cavity of Sav by simply mixing the biotinylated metal complex with Sav without further procedures. For genetic optimization purposes, recombinant Sav expressed in *Escherichia coli* (*E. coli.*) has been utilized. This facile and versatile strategy enables the screening of a set of Sav variants with various biotinylated *d*_6_-pianostool complexes. Previous studies by Ward et al. have been reviewed elsewhere [32].

To increase the throughput of the screening of metal complexes suitable for ArMs, Ward et al. attached a biotin moiety to the Cp* moiety of [Cp*IrCl_2_]_2_, which allowed screening of various bidentate ligands for the transfer hydrogenation of imines with formate or nicotinamide adenine dinucleotides NAD(P)H as hydride sources [33,34]. Relying on the same system, Rimoldi and coworkers screened the bidentate ligands **4**–**7** in the transfer hydrogenation of imine **8** (Figure 2, reaction i) [35]. Iridium cofactors **10** and **11** bearing achiral bidentate ligands afforded a racemic mixture of amine **9** in the absence of Sav, whereas those incorporated in Sav displayed some enantioselectivities (~13% ee). The chiral cofactor (*S*)-**12**, bearing the chiral bidentate ligand (*S*)-**6**, alone yielded 3% (*R*) ee in the absence of Sav. In the presence of Sav, the enantioselectivity was improved to 7% (*R*) ee for wild-type (WT) Sav and 13% (*R*) ee for the Sav(K121A) variant (Table 1, entries 1–3). In the case of (*R*)-**7**, it was found that the Sav:**13** ratio affected the enantioselectivity. The highest enantioselectivity was obtained by **13**·Sav(S112M) with a molar ratio of Sav (as a monomer):**13** = 4.0:2.5 (Table 1, entries 4–7).

Although previous studies proved the versatility of Sav-based ArMs, further improvement required drastic engineering of the Sav cavity. To this end, Ward et al. recently demonstrated three approaches: (1) encapsulating Sav within ferritin as a third coordination sphere [36], (2) introducing an additional loop in front of the cavity of Sav to prevent exposure of the abiotic cofactor to the solvent [37], and (3) connecting two Sav monomers to introduce mutations asymmetrically [38].

The third coordination sphere was installed on an artificial transfer hydrogenase, [Cp*Ir(Biot-*p*-L)Cl] **14**·Sav, by encapsulation within ferritin (Figure 2, reaction ii) [36]. Ferritin is an iron-storage protein and comprises twenty-four monomers that form a spherical cage (inner diameter 7−8 Å). The encapsulation of [Cp*Ir(Biot-*p*-L)Cl] **14**·Sav within ferritin was achieved by disassembly and re-assembly of ferritin upon acidification and neutralization in the presence of [Cp*Ir(Biot-*p*-L)Cl] **14**·Sav. In the transfer hydrogenation reaction, the protonated substrate **8** likely accessed [Cp*Ir(Biot-*p*-L)Cl] **14**·Sav through the ferritin three-fold channel, which allowed the cationic substrate to penetrate the interior. In the absence of ferritin, [Cp*Ir(Biot-*p*-L)Cl] **14**·Sav(S112A) and [Cp*Ir(Biot-*p*-L)Cl] **14**·Sav(S112K) afforded (*S*)-**9** (75% ee) and (*R*)-**9** (41% ee) (Table 1, entries 8–10). Upon encapsulation, the obtained [Cp*Ir(Biot-*p*-L)Cl] **14**·Sav@ferritin showed different enantioselectivity (Table 1, entries 12–14). Regardless of the Sav variant, [Cp*Ir(Biot-*p*-L)Cl] **14**·Sav@ferritin produced (*S*)-**9** preferentially, highlighting the influence of a third coordination sphere on the performance of ArMs.

To shield the active site from water, Ward and Woolfson et al. designed several chimeric Savs equipped with well-structured naturally occurring motifs in front of the active site for Sav-based ArMs [37]. With these chimeric Savs, transfer hydrogenation, ring-closing metathesis, and anion–π catalysis have been investigated by using corresponding abiotic cofactors. The best chimeric Savs were different depending on the reaction type. For the transfer hydrogenation of imine **15** (Figure 2, reaction iv), two chimeric Savs, with loops between the 46^th^ and 52^nd^ amino acid residues, exhibited higher catalytic activities than those of the corresponding ArMs without the loop (Table 1, entries 15–18). This suggested that these chimeric Savs are a promising starting point for further genetic optimization.

The homotetrameric nature of Sav imposes limitations on the genetic optimization of ArMs because single mutations are reflected simultaneously in all four subunits of Sav. In addition, upon incorporation of a biotinylated complex, the homotetrameric Sav affords a Poisson distribution of cofactor occupancy, which results in the stochastic placement of two abiotic cofactors next to each other. This sometimes erodes the catalytic activity. Thus, it is necessary to control the precise ratio of Sav and the biotinylated cofactor. In this context, Ward and coworkers connected two Sav monomers (Sav^A^ and Sav^B^) via a twenty-six amino acid linker (hereafter named as single-chain dimeric streptavidin, scdSav) [38]. For the precise assembly of the two scdSavs into a single quaternary structure, mutation H127C was introduced to form a disulfide bond between them. One of the two biotin-binding sites in one chain of scdSav was engineered to lose its biotin-binding capacity and give a monovalent scdSav. The versatility of this monovalent scdSav was evaluated for the transfer hydrogenation of imines with [Cp*Ir(Biot-*p*-L)Cl] **14** as a cofactor (Figure 2, reactions iii–vi). Enzyme kinetics revealed that the monovalent scdSav outperformed the corresponding divalent scdSav. This is because the monovalent scdSav provided a catalytically favored conformation of the [Cp*Ir(Biot-*p*-L)Cl] **14** complex by preventing the intrusion of a second **14** into the neighboring pocket, as clarified by the crystal structures. To improve catalytic activity, mutations were introduced at S112 and K121 in both Sav^A^ and Sav^B^ domains of scdSav (scdSav(112S^A^/121K^A^/112S^B^/121K^B^) hereafter). Under the optimized reaction conditions, monovalent scdSav(112S^A^/121A^A^/112R^B^/121K^B^) gave a TON of 17,700 with 90% (*R*) ee and a TON of 195 with 91% (*R*) ee for the reduction of **8** and **17,** respectively. For the transfer hydrogenation of **15**, scdSav(112S^A^/121K^A^/112A^B^/121A^B^) gave a TON of 1976 with 96% (*R*) ee (Table 1, entries 19–25).

In a living system, enzymatic activity is typically cross-regulated by other biochemical reactions. Inspired by concept of a zymogen, which is an inactive precursor of an enzyme, Ward and coworkers designed an artificial zymogen of the artificial transfer hydrogenase that matured with the action of a protease [39]. They also built an enzymatic reaction network causing pH change to autonomously switch the catalytic activity of the artificial transfer hydrogenases [40].

**Figure 3:**
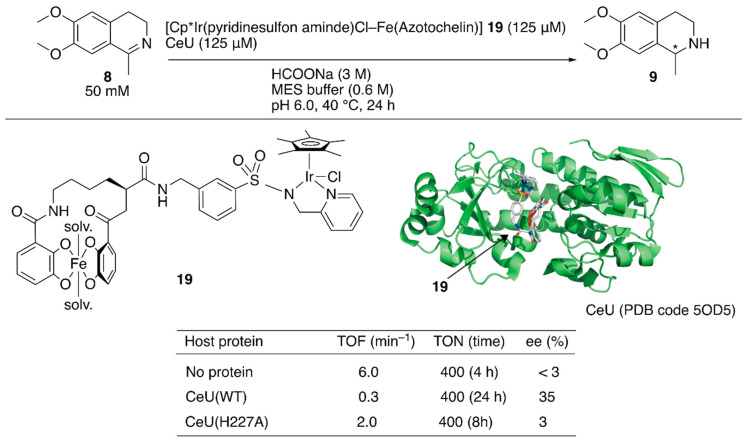
Transfer hydrogenation of an imine catalyzed by an artificial transfer hydrogenase possessing a reversibly bound iridium cofactor. Adapted from [41].

Recently, a redox switchable artificial transfer hydrogenase was reported by Duhme-Klair and Wilson et al. [41]. They focused on the redox switchable binding behavior of siderophores as a new supramolecular recognition motif for the construction of an ArM. An iron-uptake system is essential for the growth of microorganisms. Microorganisms produce siderophores that bind iron strongly and then uptake the formed iron complex via the corresponding membrane proteins. Inside the cell, the iron ion is released from the siderophore upon reduction of iron(III) to iron(II). Abiotic cofactor **19** comprising the siderophore of *Azobacter vinelanddii*, azotochelin, and a Cp*Ir complex was introduced into CeuE, an iron-siderophore periplasmic binding protein of *Campylobacter jeejuni* (Figure 3). The dissociation constant between the abiotic cofactor **19** and CeuE was determined to be 18.3 nM. Although the TOF of **19**·CeuE in the transfer hydrogenation of **8** was twenty-fold lower than that of **19** alone, some degree of enantioselectivity was observed for only **19**·CeuE (Figure 3). When His227, which was located near the iridium center, was mutated to alanine, the enantioselectivity of **19**·CeuE(H227A) decreased to 3% (*R*) ee from 35% (*R*) ee. In contrast, the TOF of **19**·CeuE(H227A) was higher than that of **19**·CeuE. These results implied that His227 coordinated to the vacant site on the iridium center and decreased the reaction rate, and this was responsible for the observed enantioselectivity. Upon addition of sodium dithionite to reduce Fe(III) to Fe(II), **19** was released from CeuE. The recovered CeuE was intact and could be reassembled with the freshly added **19** to catalyze transfer hydrogenation and afford 32% (*R*) ee with a slightly lower conversion.

#### 2.1.2. Hydrogenation

**Figure 4:**
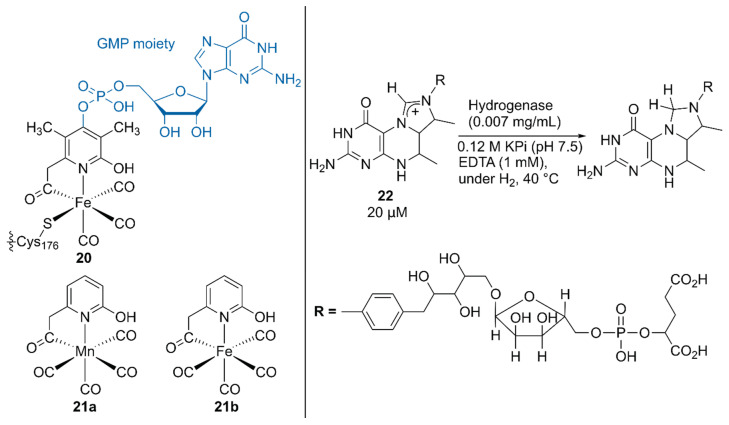
Structures of the native, synthetic Mn, and synthetic Fe cofactors of the hydrogenase. Adapted from [42].

Hu, Shima, and coworkers substituted the native cofactor of **20**·hydrogenase with the Mn model complex **21a** to obtain synthetic **21a**·hydrogenase, by using a previously established protocol for the preparation of a synthetic [Fe]·hydrogenase harboring Fe model complex **21b** (Figure 4) [42,43]. 

The higher p*K*_a_ value of the 2-OH group in **21a** than that in **21b** biased the catalysis of the synthetic **21a**·hydrogenase forward for the hydrogenation reaction of the native substrate methenyltetrahydromethanopterin (methenyl-H_4_MPT^+^) **22** with H_2_. Because a guanosine monophosphate (GMP) moiety was involved in binding the cofactor to the correct position, the lack of the GMP moiety in **21**·hydrogenase resulted in its lower activity than that of the native **20**·hydrogenase [43]. Upon normalization of the catalytic activity based on the occupancy of the cofactor, the synthetic **21a**·hydrogenase exhibited 25% higher activity than the synthetic **21b**·hydrogenase.

### 2.2. Oxidation

Dioxygen is essential for various biochemical transformations, including biosynthesis of physiologically active substances and oxidative metabolism. O_2_ is a thermodynamically powerful oxidant, but most organic molecules in the singlet state do not react with O_2_ in the triplet state. To overcome this non-reactivity, nature has utilized transition metal ions to generate metal–O_2_ complexes, as found in representative oxidases such as cytochrome P450 and methane monooxygenase. Inspired by these natural metalloenzymes, artificial oxidases have been developed to catalyze various oxygen insertion reactions, including peroxidation, sulfoxidation, epoxidation, dihydroxylation, and C–H oxidation. Artificial cofactors used in this chemistry can be classified into three types: (1) transition metal ions, such as Os (e.g., metal source = K_2_[OsO_2_(OH)_4_]), V (e.g., metal source = VOSO_4_, Na_3_VO_4_), and Mn (e.g., metal source = MnCl_2_, Mn(acetate)_2_), directly coordinated to the amino acid residue, (2) Fe^3+^/Mn^3+^–porphyrinoids replaced with the native cofactor of hemoproteins, and (3) non-heme Fe^3+^/Mn^3+^ complexes covalently conjugated with the protein [7]. In this section, recent progress in the field of artificial oxidases is presented.

**Figure 5:**
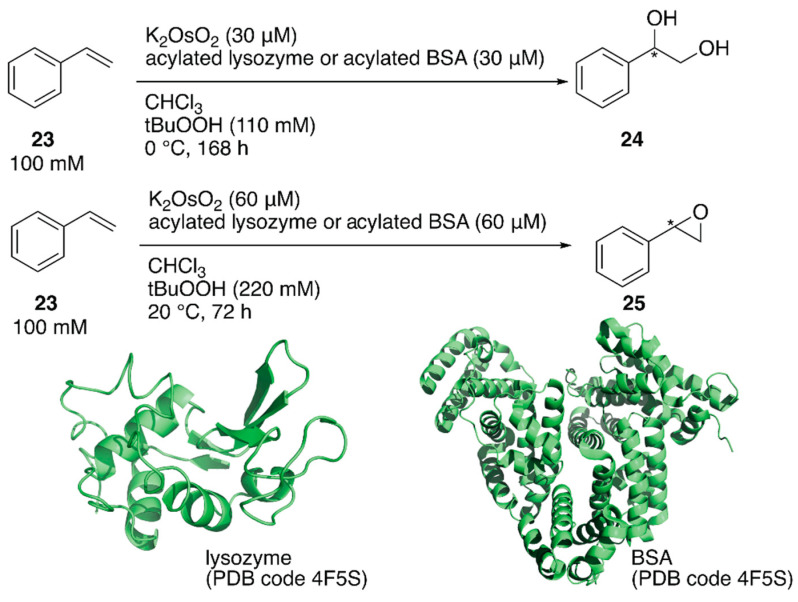
Oxidation reactions catalyzed by acylated protein-bearing Os ions in organic solvents. Adapted from [47].

ArMs catalyzing dihydroxylation, that is, artificial dihydroxylases, have been constructed from osmium ions and bovine serum albumin (BSA) or Sav by Kokubo et al. and Ward et al., respectively [44,45]. Following these examples, Tiller et al. constructed an artificial dihydroxylase from K_2_OsO_2_(OH)_4_ and laccase, the amino group of which was conjugated with poly(2-methyl-oxazoline), to use ArMs in organic solvents. The obtained polymer conjugate was soluble in organic solvents and catalyzed the enantioselective dihydroxylation of styrene in chloroform [46]. It was reasoned that the observed enantioselectivity was attributable to blocking of the primary amino groups in the protein to prevent non-specific osmium binding. Inspired by this, instead of polymers, *N*-acetylated (AA), *N*-propionylated (PA), and *N*-hexanoylated (HA) lysozyme and BSA were recently prepared as protein scaffolds of the ArM for enantioselective oxidation [47]. Although these modified proteins are not soluble in organic solvents, they form stable suspensions. When an enzyme is utilized in organic solvents, in general, its protonation state affects the catalytic activity. Therefore, the artificial oxidases were adjusted to the optimized pH value in aqueous solution before lyophilization. *N*-Acylated proteins were mixed with K_2_OsO_2_ in a metal:protein ratio of 1:1 and tested for the enantioselective dihydroxylation of styrene **23** (Figure 5). Os·lysozymes displayed 98% ee (TON = 289), 94% ee (TON = 85), and 55% ee (TON = 103) with HA, PA, and AA modification, respectively (Table 2). Epoxidation of styrene **23** was also tested by using *N*-acylated proteins mixed with RuCl_3_ (Figure 5). When non-*N*-acylated lysozyme (= native lysozyme) was used, the Ru·lysozyme afforded 10% ee with TON = 361. In contrast, improved enantioselectivities were observed with *N*-acylated artificial oxidases (Table 2). The highest activity (82% ee with TON = 2613) was obtained by using Ru·HA-BSA, which was mixed in a metal:protein ratio of 3:1, indicating that there were three confined metal-binding sites in BSA.

Fujieda and Itoh et al. also demonstrated dihydroxylation with a cupin superfamily protein (TM1459) as a macromolecular ligand of the osmium ion. TM1459 is a highly thermally stable homodimeric Mn^2+^-binding protein with a molecular mass of 13 kDa. It binds to a manganese ion with four histidine residues located in the β-barrel structure. On substituting the native manganese ion with an osmium ion (K_2_[OsO_2_(OH)_4_] as the metal source), the resulting Os^3+^·TM1459 regioselectively catalyzed the *cis*-1,2-dihydroxylation reaction of an alkene with hydrogen peroxide as the terminal oxidant [48].

**Figure 6:**
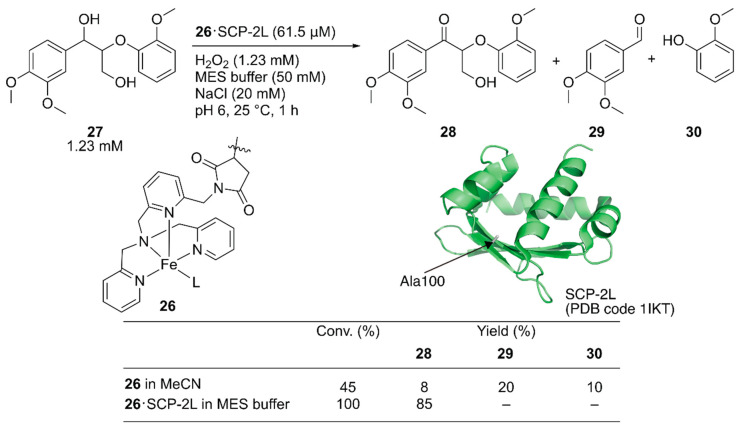
Selective oxidation of the lignin model compound by artificial oxidase **26**·SCP-2L. Adapted from [49].

Jarvis and coworkers covalently incorporated the Fe-tris(pyridylmethyl)amine (tpa) complex **26** at the mutationally introduced cysteine residue (A100C) of steroid carrier protein 2L (SCP-2L) [49]. Previously, Mahy et al. covalently conjugated a similar iron complex with β-lactoglobulin (βLG) to catalyze enantioselective sulfoxidation [50]. Jarvis et al. tested the obtained ArM for the selective oxidation of the benzylic alcohol of lignin β-*O*-4 model compound **27** to form **28**. Full conversion was achieved with **26**·SCP-2L without any formation of the β-*O*-4 cleaved products **29** and **30**, whereas cofactor **26** alone gave a mixture of the three compounds (Figure 6).

### 2.3. C–C Bond Formation

To build a framework of organic molecules, the C–C bond forming reaction is very useful in synthetic chemistry. Following significant developments in C–C bond formation catalyzed by metal complexes, ArM technology has allowed such complexes to be utilized in water and display chemoselectivity. The reported ArM-catalyzed C–C bond formations include allylic alkylation, Suzuki cross-coupling, Heck reaction, C–H activation, olefin metathesis, polymerization, Friedel–Crafts reaction, Diels–Alder reaction, and cyclopropanation. In this section, we summarize the recent progress in the field of C–C bond formation catalyzed by ArMs.

#### 2.3.1. Cyclopropanation

Arnold et al. and Fasan et al. respectively utilized directed evolution of hemoproteins to achieve carbene insertion into olefins (cyclopropanation) and nitrene insertion into C–H bonds by using cytochrome P450 and myoglobin (Mb) [51,52]. Instead of heme as the active site, Lewis and coworkers employed an ArM comprising a dirhodium cofactor and a prolyl oligopeptidase scaffold from *Pyrococcus furiosus* [53]. The genetically optimized dirhodium ArM catalyzed enantioselective cyclopropanation. In this section, recent reports of ArM-catalyzed cyclopropanation are summarized.

**Figure 7:**
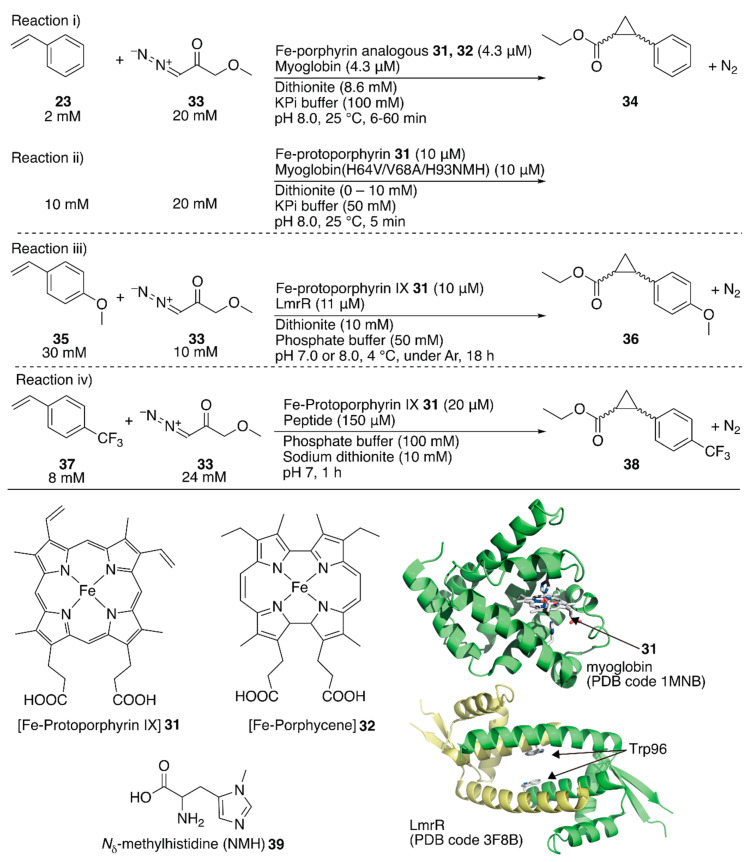
Cyclopropanation catalyzed by ArMs containing iron porphyrinoids as their cofactor. Adapted from [55,58,59,62].

The replaceability of the native cofactor of Mb, Fe-protoporphyrin IX **31** (Figure 7), with a synthetic planar metal complex has made Mb an attractive protein scaffold for ArMs. Watanabe et al. and Hayashi et al. developed various ArMs catalyzing H_2_O_2_-dependent oxidation reactions. Their previous research on oxidation chemistry with hemoprotein-based ArMs has been reviewed elsewhere [7,12,54]. In contrast to the studies on the directed evolution of hemoproteins, Hayashi, Lehnert, Hasegawa et al. enabled Mb to catalyze cyclopropanation by replacing Fe-protoporphyrin IX **31** with Fe-porphycene **32** without any mutation (Figure 7) [55]. They focused on porphycene, which is a constitutional isomer of porphyrin with different physicochemical properties and reactivities, as they had previously found that Mb reconstituted with Mn-porphycene catalyzes the hydroxylation of an inert C–H bond [56,57]. It was found that **32**·Mb catalyzed the cyclopropanation of styrene **23** with ethyl diazoacetate **33** with a TON of 133 in the presence of dithionite, whereas with **32** alone, the TON was 9 (Figure 7, reaction i; Table 3, entries 3 and 4). Kinetic analysis revealed that the catalytic efficiency *k*_cat_/*K*_M_, where *k*_cat_ is the catalytic rate constant and *K*_M_ is the Michaelis constant, of **32**·Mb was twenty-six-fold higher than that of the native **31**·Mb. The diastereomeric excess (de) for the (*E*)-conformer of **34** for **32**·Mb was >99%, whereas those for **31**, **32**, and **31**·Mb were 62, 85, and 53%, respectively (Table 3, entries 1–4). As an alternative approach to directed evolution and cofactor replacement mentioned above, Hilvert and coworkers demonstrated that the introduction of unnatural amino acid **39** as the heme axial ligand of Mb also allowed cyclopropanation (Figure 7, reaction ii). The details are described in the following section [58].

Roelfes et al. utilized an *E. coli* recombinant lactococcal multidrug resistance regulator (LmrR) as a host protein for various ArMs. LmrR is a homodimeric protein with a size of 13.5 kDa per monomer. A unique flat hydrophobic cavity formed at the interface of the dimer allows the binding of flat aromatic organic molecules and planar metal complexes [11]. As mentioned above, the rapid growth of hemoprotein engineering toward cyclopropanation prompted them to insert heme **31** into LmrR (Figure 7) [59]. The crystal structure of **31**·LmrR revealed that heme **31** was located between the two W96 residues and was expected to be shielded from other external molecules such as substrates (Figure 7). The electron density of heme **31** also suggested that there were several binding modes of heme **31** in LmrR. Accordingly, considerable dynamics in the heme-binding may exist, allowing conformational changes in LmrR during the catalytic reaction. This was supported by molecular dynamics (MD) simulations indicating that one of the two W96 residues was rotated toward the outside of the pore to create a space around the heme for substrate access. The cyclopropanation of **35** with **33** was investigated (Figure 7, reaction iii). Although **31**·LmrR displayed a TON of 247 with 17% ee, a TON of 51 with no ee was observed for **31** alone (Table 3, entries 5 and 6). This result demonstrates that the LmrR scaffold accelerated the reaction and improved its selectivity. The effect of the hydrophobic pores comprising M8, V15, F93, W96, and D100 was investigated by introducing alanine mutations (Table 3, entries 7–11). The absence of enantioselectivity for **31**·LmrR(W96A) was attributed to the loosening of the chiral interaction between **31** and LmrR. The highest activity was obtained with **31**·LmrR(M8A) when the reaction was performed at pH 7.0 (Table 3, entry 12).

Korendovych and coworkers found that metal-binding amyloidogenic short peptides displayed hydrolytic activity [60,61]. Recently, they installed a binding site for Fe-protoporphyrin IX **31** on a β-sheet amyloid for the cyclopropanation reaction (Figure 7, reaction iv) [62]. From a series of seven-residue peptides they have prepared, the peptide, whose sequence is LILHLFL, (hereafter LILHLFL peptide) forms a β-sheet amyloid and binds to Fe-protoporphyrin IX **31** in a peptide monomer:Fe-protoporphyrin IX **31** ratio of 4:1 (*K*_d_ = 2 μM). The circular dichroism (CD) signal indicated that the β-sheet amyloid provided a chiral environment around Fe-protoporphyrin IX **31**. The resulting ArM catalyzed cyclopropanation of **33** and **37** to afford a TON of 208 and with 12% ee for *cis-***38** and 40% ee for *trans-***38**, whereas a decreased TON and no enantioselectivity were observed in the case of Fe-protoporphyrin IX **31** alone (Table 3, entries 13–16). The LHLH(l-NMe)FL peptide, which has a methylated backbone nitrogen atom, does not form the β-sheet structure, although it still binds to Fe-protoporphyrin IX **31** to some extent. No enantioselectivity was obtained with this peptide, confirming the importance of the higher-ordered supramolecular structure. Additionally, the enantiomer of the LILHLFL peptide, (d)-LILHLFL peptide, was found to give almost exactly opposite enantioselectivity to the LILHLFL peptide, whereas the TON remained almost the same.

#### 2.3.2. Friedel–Crafts Reaction

**Figure 8:**
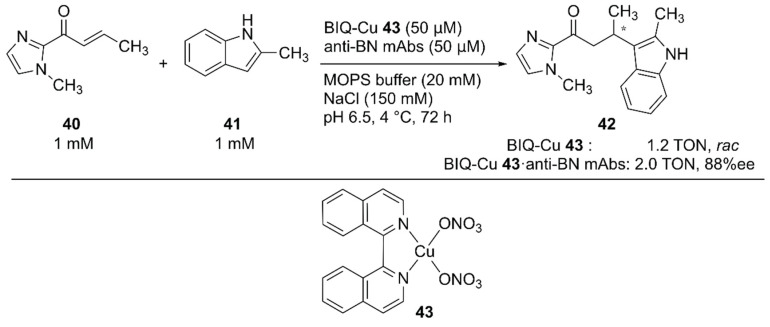
Friedel–Crafts reaction catalyzed by BIQ-Cu **43**·anti-BN mAbs. Adapted from [66].

For the Friedel–Crafts reaction, Roelfes and coworkers previously developed LmrR-based ArMs [63,64,65]. Recently, Harada and coworkers employed monoclonal antibodies (mAbs) as protein scaffolds for ArMs catalyzing Friedel–Crafts reactions [66]. They previously developed atroposelective mAbs by immunological optimization of mAbs over binaphthyl (BN) derivatives [67,68]. These anti-BN mAbs could bind 1,1′-bi-isoquinoline (BIQ)-based metal catalysts and provide a chiral environment for the selective reaction. Friedel–Crafts alkylation reactions of **40** and **41** to yield **42** were investigated in the presence of anti-BN mAbs and BIQ-Cu **43** (Figure 8). Upon complexation of BIQ-Cu **43** with mAb R44E1, 88% ee ((+)-**42**) with 10% yield was obtained. The authors assumed that the obtained enantioselectivity corresponded to 99% ee when the contribution of unbound BIQ-Cu **43** was excluded.

#### 2.3.3. Michael Addition Reaction

**Figure 9:**
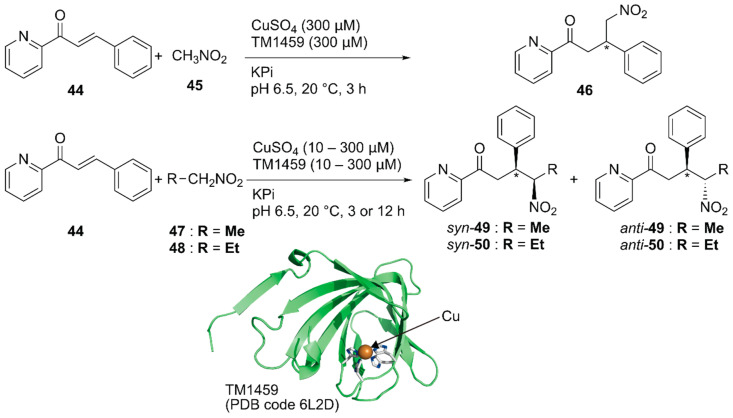
Michael addition of nitroalkanes to azachalcones catalyzed by Cu-TM1459. Adapted from [70].

Previously, Roelfes and coworkers reported a DNA-based ArM as a catalyst for the Michael addition reaction [69]. Recently, Fujieda et al. developed a protein-based ArM for this transformation [70]. As described above, their previous work on catalytic dihydroxylation with Os·TM-1459 proved that TM-1459 could be utilized as a ligand for metal ions [48]. This finding prompted them to prepare Cu·TM1459 for the stereoselective Michael addition of nitroalkanes. Based on the crystal structure of Cu·TM-1459(WT), a small library of TM-1459 variants bearing copper-binding sites comprising two or three histidine residues were prepared in silico. The library was tested for the Michael addition of nitromethane **45** to azachalcone **44** (Figure 9). 

Although the copper ion alone and Cu·TM-1459(WT) afforded racemic product **46**, Cu·TM-1459(H52A) with a 3-His binding site and Cu·TM-1459(H54A/H58A) with a 2-His binding site gave 99% (*S*) ee and 89% (*R*) ee, respectively. C106 of Cu·TM-1459(H52A) and F104 of Cu·TM-1459(H54A/H58A) were found to be crucial for the enantioselectivity. This is consistent with the results of the docking simulation of azachalcone **44** for both variants. When 0.1 mol% catalyst was used for the Michael addition of nitroethane **47** to azachalcone **44**, *syn*-selective product **49** was obtained with a highest TON of 250 and 97% ee. Both Cu·TM-1459(H52A) and Cu·TM-1459(H54A/H58A) preferentially afforded *syn*-product **50** with the opposite enantioselectivity. Unlike other variants, only Cu·TM-1459(H52A/C106N) favored the *anti*-product **50**. It was thus demonstrated that the Cu·TM-1459 variants controlled the *syn* or *anti* preference in the Michael addition with enantioselectivity.

#### 2.3.4. Diels–Alder Reaction

Inspired by the early example of an ArM for the Diels–Alder reaction, which was reported by Reetz et al. [71], several groups have developed artificial Diels-Alderases [72,73,74,75,76,77,78]. In most cases, copper was employed as a metal center bearing polypyridyl ligands.

**Figure 10:**
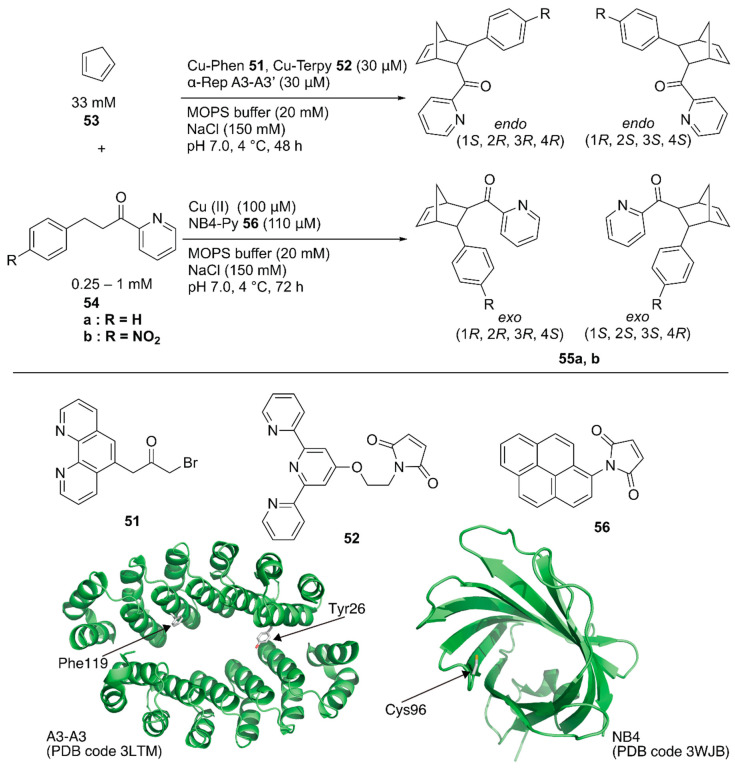
Artificial copper enzymes as catalysts for the Diels–Alder reaction. Adapted from [79,83].

Recently, Ricoux and coworkers employed a bidomain α-selenoid repeat protein (α-Rep A3) as a scaffold for an artificial Diels-Alderase [79]. α-Rep A3 forms a dimer (A3-A3) with a confined cavity. The two domains of α-Rep A3 were genetically linked to form a single chain A3-A3′ and one cysteine residue was introduced into A3-A3′ (Y26C or F119C), enabling 1:1 ligand anchoring in one protein cavity. Polypyridyl copper complexes, Cu-phenanthroline (Phen) **51** or -terpyridine (Terpy) **52**, were covalently attached to Y26C or F119C of A3-A3′ (Figure 10). As a benchmark reaction, the Diels–Alder reaction between 2-azachalcone **54a** and cyclopentadiene **53** was tested. ArM **51**·A3-A3′ generally resulted in a higher conversion than **52**·A3-A3′ because of the larger cavity size. In contrast, **52**·A3-A3′ exhibited higher enantioselectivity, with up to 52% ee (Table 4, entries 1–4).

Hayashi and coworkers covalently linked *N*-(1-pyrenyl)maleimide **56** into the hydrophobic cavity of an *E. coli* recombinant nitrobindin (NB) with a Cys96 mutation to form a π-extended cavity in the NB. Naturally, NB is a hemoprotein with a size of 19 kDa. They removed the heme-binding site in the NB(WT) by mutation and utilized the obtained NB mutant as a protein scaffold [80,81,82]. The cavity of NB·**56** acted as a platform for substrate binding for the copper ion-catalyzed Diels–Alder reaction between azachalcone **54** and cyclopentadiene **53** (Figure 10) [83]. When the NB variant NB4, with a small cavity size was used, 94% conversion with *endo*/*exo* selectivity of 96/4 and 78% ee was obtained as the best result (Table 4, entries 5–8). Based on CD measurements, MD calculations, and docking simulations, NB4·**56** was suggested to have a properly shaped cavity for good selectivity.

#### 2.3.5. Aldol Condensation

**Figure 11:**
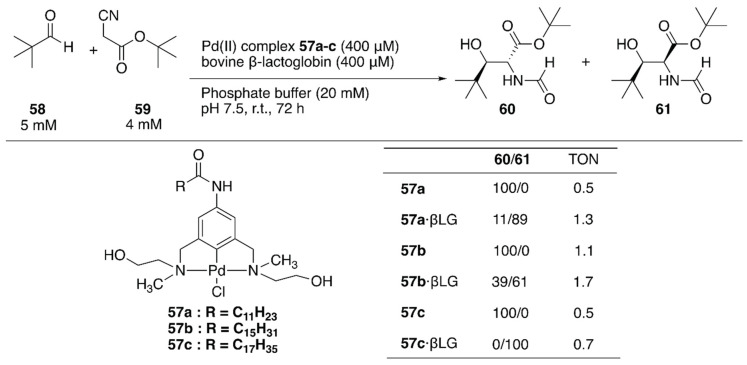
Aldol condensation of *tert*-butyl isocyanoacetate and pivalaldehyde catalyzed by ArMs containing a palladium center. Adapted from [86].

Two decades after the reports of metal-free artificial aldolases based on antibodies [84,85], Thorimbert et al. introduced the prochiral Pd(II)·NCN-pincer complexes **57a**–**c** into bovine β-lactoglobulin (βLG) by utilizing supramolecular recognition between βLG and a fatty acid (Figure 11) [86]. A molecular docking simulation predicted that a hydroxy group installed on a substituent of the nitrogen atom of the NCN ligand would be beneficial for a stable supramolecular assembly because of the *H*-bond interaction between the OH group and the carboxylate of Asp85 in βLG. In the absence of βLG, an aldol condensation reaction between **58** and **59** catalyzed by **57a**–**c** alone yielded exclusively the *trans* product **60** (Figure 11). In contrast, **57a**–**c**·βLG preferentially gave *cis* product **61**; this is probably because the stereo-configurations of **57a**–**c** were defined by the βLG matrix, as suggested by the molecular docking simulation.

### 2.4. Unnatural Amino Acids

As described in the next section on the directed evolution of ArMs, mutagenesis is still a powerful tool for expanding substrate scope, improving selectivity, and increasing the reaction rate. Unnatural catalytic functionality can even be installed into natural enzymes by directed evolution [87,88]. However, only naturally occurring amino acids can be selected in the typical mutagenesis. Owing to the rapid development of genetic code reprogramming [89,90], artificial enzymes can be constructed by genetically incorporating unnatural amino acids into proteins [23]. In this section, we describe the recent developments in the field of artificial enzymes containing genetically encoded unnatural amino acids as the active site.

**Figure 12:**
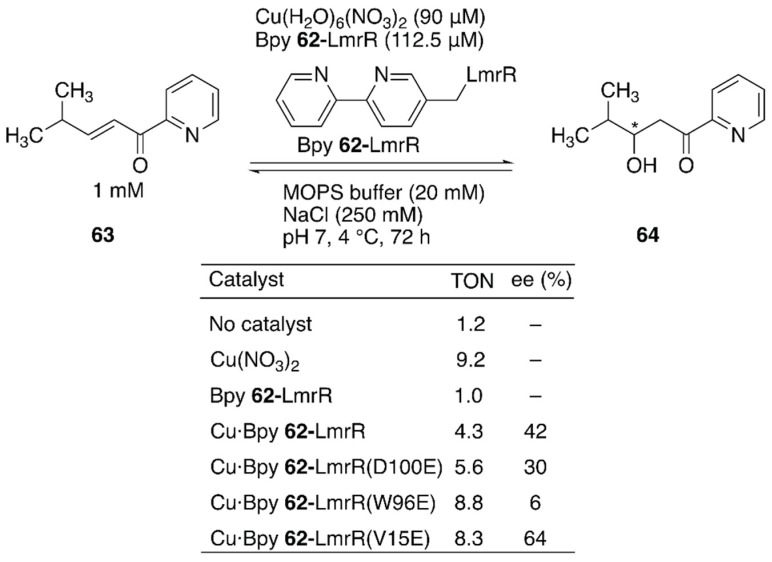
Enantioselective hydration reaction catalyzed by Cu**·**Bpy **62**-LmrR. Adapted from [91]. See Figure 7 for the crystal structure of LmrR.

Enantioselective hydration of enones is challenging owing to difficulties in controlling water, which is small and less reactive at neutral pH, as a nucleophile (Figure 12). To overcome this challenge, Maréchal et al. constructed an ArM to catalyze this reaction by genetically incorporating an unnatural amino acid, (2,2′-bipyridine-5yl)alanine (BpyA) **62**, as a copper-binding site into LmrR [91].

The authors demonstrated that a sequential combination of cluster model calculations, protein–ligand docking, and MD enabled the identification of catalytically active substrate–cofactor–host protein geometry. A unique flat-shaped hydrophobic cavity formed at the interface of the dimeric LmrR was thought to facilitate substrate binding. The M89 residue of LmrR, which is located at the end of the cavity, was selected for the incorporation of BpyA to avoid the formation of the metal complex with a copper:ligand ratio of 1:2. Initially, a model structure comprising a Cu(II) (2,2′-bipyridine) complex, a carboxylate as a general acid–base catalyst, substrate **63**, and water was optimized with density functional theory (DFT) calculations to estimate the favorable position of the carboxylate. With the calculated structure and the model structure of Bpy **62**-LmrR, protein–ligand docking experiments were performed to find possible positions to place the carboxylate residues in LmrR. D100, V15, and W96 were found to be potential candidates for replacement with glutamic acid. As predicted by MD analysis of these variants, the Bpy **62**-LmrR(V15E) variant gave the highest enantioselectivity among the three Bpy **62**-LmrR variants (Figure 12).

**Figure 13:**
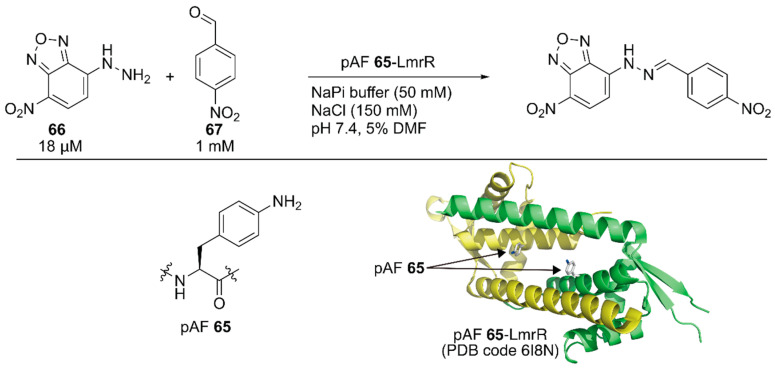
Hydrazine formation catalyzed by an artificial enzyme containing an unnatural amino acid at the active site. Adapted from [92].

Subsequent to the development of the above ArM containing an unnatural amino acid, Roelfes and coworkers developed a nonmetal artificial enzyme by incorporating a catalytically active unnatural amino acid [92]. They introduced the *p*-phenylalanine (pAF) **65** residue to LmrR by combining an expanded genetic code method for *p*-azidophenylalanine incorporation with subsequent chemical reduction. The catalytic activities of the artificial enzyme toward hydrazine formation between **66** and **67** were evaluated (Figure 13). The reaction proceeded with 6% yield in the absence of the catalyst, and 1 mM aniline boosted the reaction to 40% yield. Owing to the recruitment of the substrates in its hydrophobic cavity, even 10 μM LmrR alone was found to accelerate the reaction to give the product in 46% yield. Further improvement was observed with LmrR(V15pAF), which provided the best result (72% yield) among the four pAF **65**-containing LmrR variants. A related oxime formation reaction was also tested and LmrR(V15pAF) exhibited satisfactory performance, highlighting its proficient activity as a general nucleophilic catalyst. Mayer, Roelfes, and coworkers applied directed evolution to LmrR(V15pAF) for hydrazine formation [93]. They targeted 13 residues forming the hydrophobic pore of LmrR(V15pAF) and conducted several rounds of evolution to identify beneficial mutations. Combination of the beneficial mutations finally achieved the LmrR_pAF_RMHL variant, in which four additional mutations were incorporated into LmrR(V15pAF), displaying a *k*_cat_ value 91-times higher than that of its parent. Afterwards, an ArM based on LmrR(V15pAF) was developed, as described later in this review [94].

The electronic properties of heme **31** in a protein scaffold are controlled by the amino acid residue acting as an axial ligand, leading to various catalytic reactions, including oxidation and carbene transfer (Figure 7). Hilvert and coworkers added an unnatural amino acid, *N*_δ_-methylhistidine (NMH) **39**, to the repertoire of axial ligands of hemoproteins (Figure 7). Introducing NMH **39** into Mb resulted in improved peroxidase activity [95]. Subsequent directed evolution enabled Mb to display peroxidase activity comparable to that of natural peroxidase enzymes. This is because *N*-methylation led to breaking of the hydrogen bond between the axial heme ligand NMH **39** and Ser92, which increased the electrophilicity of the iron center and facilitated the reaction with H_2_O_2_.

This result prompted them to test Mb(H93NMH) for a carbene transfer reaction [58]. Two additional mutations, H64V and V68A, were introduced into Mb(H93NMH) because these mutations were previously found to be beneficial for the carbene transfer reaction [52,96,97]. According to the crystal structure of Mb(H64V/V68A/H93NMH), hydrogen bonding was not observed between NMH93 and Ser92 and the imidazole ring of NMH was rotated by ~100° relative to His93 in Mb(H64V/V68A). These changes resulted in small structural adjustments in the proximal pocket and a widened entrance of the distal pocket, allowing efficient access of ethyl diazoacetate **33** to the active site of Mb. Moreover, these structural changes affected the redox potential of heme. The redox potential of Fe^3+^/Fe^2+^ for Mb(H64V/V68A/H93NMH) was 77 mV (vs. normal hydrogen electrode), whereas that for Mb(H64V/V68A) was 30 mV, confirming the increased electrophilicity of the iron center. A cyclopropanation reaction with styrene **23** and ethyl diazoacetate **33** was tested in the presence of dithionite and anaerobic conditions. The activity of Mb(H64V/V68A/H93NMH) was comparable to that of Mb(H64V/V68A) (Figure 7). When less dithionite was used, the TON of Mb(H64V/V68A) decreased, whereas Mb(H64V/V68A/H93NMH) maintained its catalytic activity. Even in the complete absence of dithionite, Mb(H64V/V68A/H93NMH) afforded a TON of ~700 within 5 min. Furthermore, Mb(H64V/V68A/H93NMH) gave a TON of ~800 within 7 h in air without dithionite, indicating its tolerance of O_2_. In contrast, Mb(H64V/V68A) did not show any significant activity under the same conditions.

## 3. Cascade, Sequential, and Synergetic Reactions

From the viewpoint of sustainable chemistry, a multistep transformation enabled by multiple catalysts in one pot is quite attractive. This is because it can improve the overall yield and reduce the use of chemicals such as organic solvents by omitting laborious isolation of the reaction intermediates. In particular, the cooperative use of an enzyme with a metal complex catalyst allows the combination of the high reaction selectivity of enzymes with the reaction repertoire of metal complexes. However, this is challenging because these two types of catalysts are often mutually inactivated and the reaction conditions for one cannot be applied to the other [98,99,100]. Recently, it was found that ArMs allow concurrent use of metal complex with other catalysts, such as an enzyme or another ArM [24,34,40,101]. In this section, we present recent works on sequential transformations using ArMs.

Alcohol dehydrogenases (ADHs) catalyze the reduction of a ketone to the corresponding chiral alcohol and are therefore valuable tools for synthetic chemistry. Owing to the high cost of NAD(P)H, it is necessary to use an in situ NAD(P)H regeneration system. Previously, Ward et al. constructed NAD(P)H and synthetic NADH regeneration systems by using Sav-based ArMs and coupled them with several natural enzymes [24,101].

**Figure 14:**
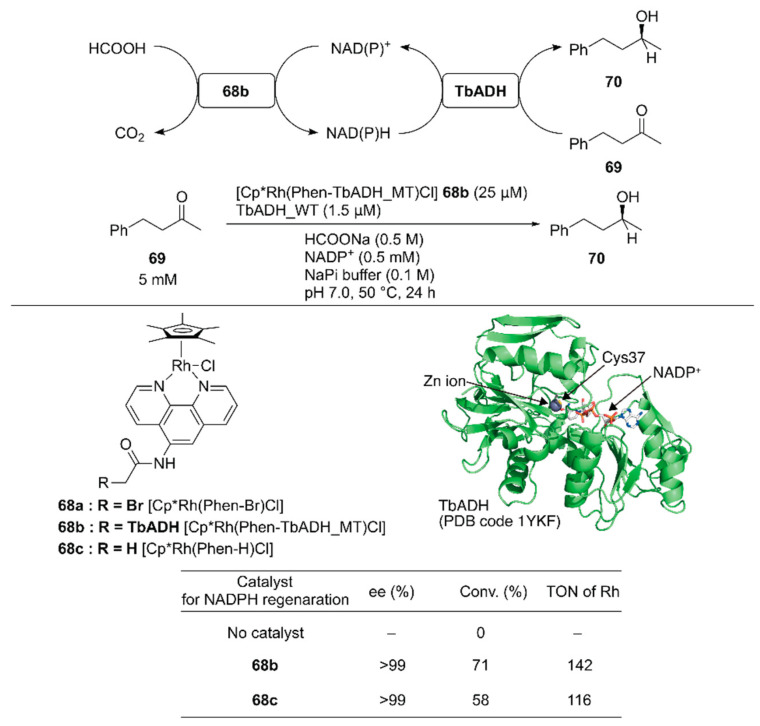
Reduction of 4-phenyl-2-butanone **69** by TbADH-based ArM **68b** coupled with TbADH. Adapted from [102].

Pordea and coworkers constructed an ADH-based ArM that catalyzed NAD(P)^+^ reduction with formate as a hydride source to couple with the native catalytic activity of ADH toward ketone reduction [102]. Because of its stability at relatively high temperatures (up to 65–86 °C) and in the presence of organic solvents, an ADH from the thermophilic species *Thermoanaerobacter brockii* (TbADH) was selected. The active site of the TbADH(WT) contains the catalytic zinc ion coordinated with C37, H59, and D150. To place the abiotic cofactor Cp*Rh(Phen-Br) **68a** in the proximity of the NAD(P)H redox site, it was conjugated at C37 of ADH. To remove the native zinc-binding site of ADH and avoid undesired conjugation of **68a**, five mutations H59A/D150A/C203S/C283A/C295A were introduced into TbADH (hereafter, TbADH_MT). After covalent modification of TbADH_MT with **68a**, the resulting **68b** catalyzed the reduction of NADP^+^ with TOF = 74 h^−1^. A chemoenzymatic cascade involving the reduction of 4-phenyl-2-butanone **69** by TbADH(WT) and NADPH regeneration catalyzed by **68b** was tested (Figure 14). Although the free catalyst **68c** displayed a higher TOF (207 h^−1^) than **68b** in the NADP^+^ reduction reaction alone, **68b** gave a higher conversion than **68c** in the case of the chemoenzymatic cascade. This result indicates that compartmentalization of the rhodium complex diminishes the mutual deactivation of both the rhodium complex and TbADH. Owing to the enantioselectivity of TbADH(WT), the (*S*)-enantiomer **70** was obtained from this chemoenzymatic cascade.

In 2011, the first ArMs catalyzing olefin metathesis, artificial metathases, were reported by Ward and Hilvert, respectively [103,104]. As the abiotic cofactor, a second-generation Grubbs–Hoveyda (GH) catalyst possessing an anchoring moiety was utilized in these studies, as well as in subsequent reports from other research groups [105,106,107,108,109,110].

**Figure 15:**
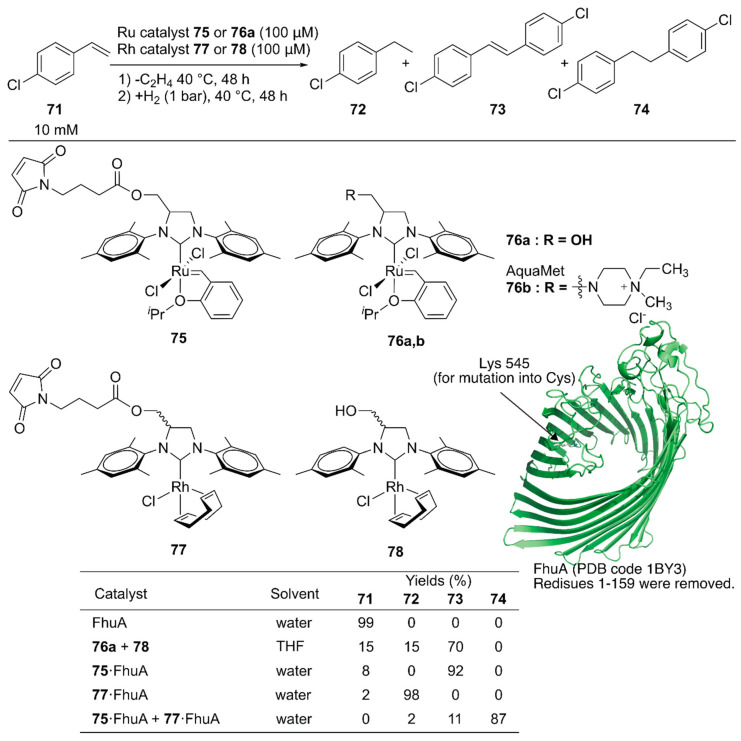
Cascade reactions involving olefin metathesis and subsequent hydrogenation catalyzed by Ru and Rh ArMs. Adapted from [111].

Schwaneberg and coworkers developed a cascade reaction comprising two different ArMs [111]. The GH catalyst **75** catalyzing cross-metathesis and Rh catalyst **77** catalyzing hydrogenation reaction were individually covalently anchored on ferric hydroxamate uptake protein component A (FhuA), to give ArMs **75**·FhuA and **77**·FhuA (Figure 15). The benchmark reaction produced **74** via cross-metathesis of chlorostyrene **71**, yielding stilbene derivative **73**, which subsequently underwent hydrogenation to form **74**. A homogeneous mixture of the GH catalyst **76a** and Rh catalyst **78** in tetrahydrofuran (THF) resulted in 70% yield of **73**, suggesting low hydrogenation efficiency. In contrast, the reaction efficiently produced **74** in 87% yield when ArMs **75**·FhuA and **77**·FhuA were used. This result demonstrated that ArM technology is beneficial when two metal catalysts are combined by compartmentalization within a protein scaffold.

**Figure 16:**
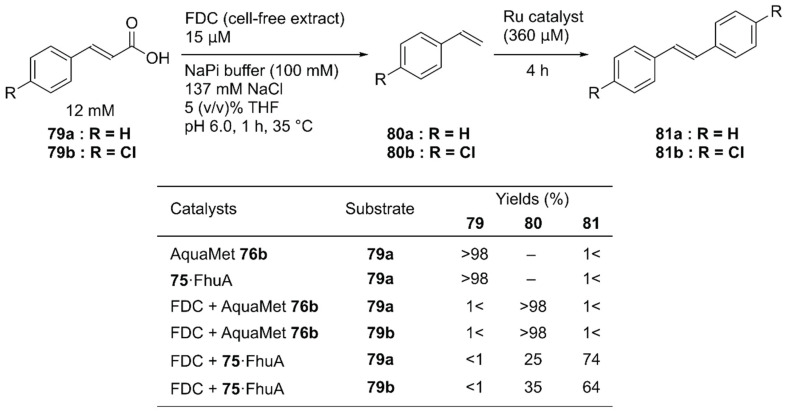
Sequential one-pot decarboxylation and olefin metathesis catalyzed by an FDC enzyme and a GH catalyst. Adapted from [112]. See Figure 15 for the structure of FhuA.

Instead of concurrent cascade reactions, the authors also reported a sequential one-pot reaction involving ferulic acid decarboxylase (FDC) and **75**·FhuA [112]. The cinnamic acid derivative **79** was first decarboxylated by FDC to produce styrene derivatives **80**; this was followed by cross-metathesis catalyzed by **75**·FhuA to produce symmetric stilbene derivatives **81** (Figure 16). To maintain FDC activity, the GH catalyst and **75**·FhuA were added after the first decarboxylation step was completed. Because FDC was used as a cell-free extract, the commercially available water-soluble GH catalyst, AquaMet **76b**, was inactivated. In the case of **75**·FhuA, however, stilbene derivatives **81a** and **81b** were obtained in 74% and 64% yield, respectively.

Ferrer, Guallar and coworkers created a unique artificial enzyme with two active sites, one of which comprised amino acid residues and the other was modified with a metal complex [113]. First, an additional catalytic site **88** was introduced into a serine ester hydrolase, EH1, to form the enzyme **88_**EH1 with two active sites, which was termed as a plurizyme. **88**-EH1 exhibited increased *k*_cat_ and *k*_cat_/*K*_m_ values, stereoselectivity, and temperature tolerance for ester hydrolysis, indicating the advantage of harboring multiple enzymatic sites.

**Figure 17:**
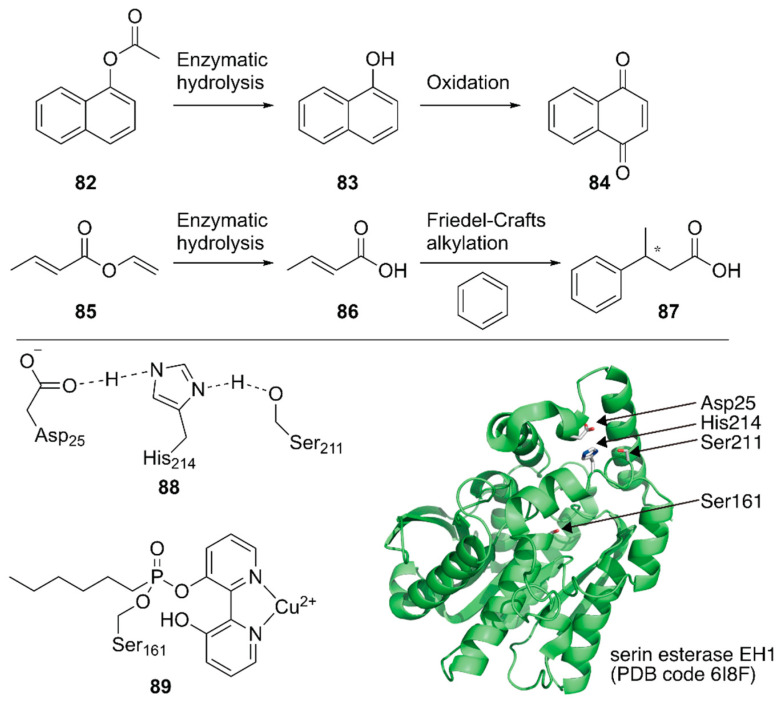
Chemoenzymatic cascade reactions catalyzed by an artificial enzyme with two active sites. Adapted from [113].

Next, the natural enzymatic site of **88_**EH1 was selectively modified by a bipyridine-incorporating suicide inhibitor that could covalently link to catalytic Ser161; it was further treated with Cu^2+^ to construct ArM **89**·**88**-EH1 with one hydrolysis bio-site and one copper chemo-site. **89**·**88**-EH1 was applied to two cascade reactions, namely, the hydrolysis of an ester at **88** followed by an oxidation or Friedel–Crafts alkylation reaction at **89** (Figure 17). For both reactions, **89**·**88-**EH1 gave efficient conversions, with 99% ee for (*S*)-3-phenylbutyric acid in the latter reaction.

**Figure 18:**
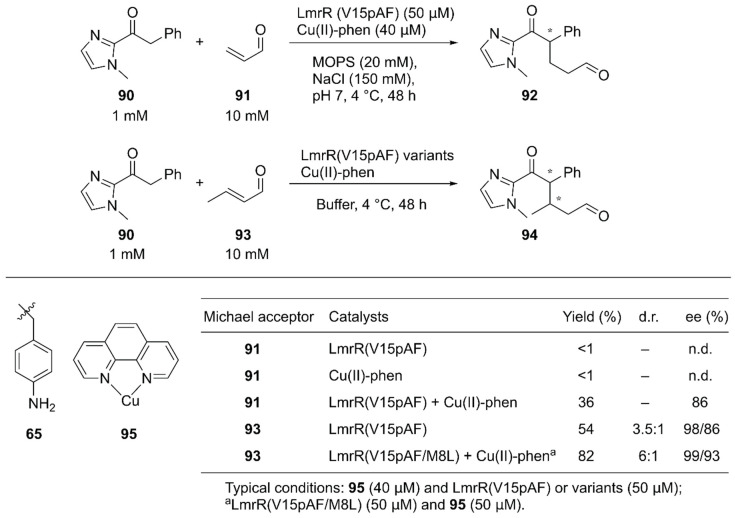
Synergistic catalysis by the unnatural amino acid **65** and Cu complex **95** in a LmrR-based artificial enzyme. Adapted from [94]. See Figure 13 for the crystal structure of LmrR (V15pAF).

Roelfes and coworkers reported synergistic catalysis by an ArM harboring two abiological catalytic sites [94]. Into a dimeric protein LmrR with a large hydrophobic cavity, they simultaneously introduced the unnatural catalytic pAF **65** residue and the Lewis acidic Cu(II)-Phen complex **95** by utilizing supramolecular interactions with W96/W96′ of LmrR [63] (Figure 13, Figure 18). Initially, the Michael addition reaction between ketone **90** and acrolein **91** was demonstrated (Figure 18). Although neither the LmrR(V15pAF) variant nor Cu(II)-Phen **95** produced any product when used separately, the combination of these two catalysts inside a protein scaffold synergistically promoted the reaction to produce **92** in 36% yield with 86% ee. Next, crotonaldehyde **93** was used as a Michael acceptor to obtain **94** with two chiral centers. When the LmrR(V15pAF) variant and Cu(II)-Phen **95** were combined, excellent enantioselectivities for both diastereomers with 98% and 86% ee, respectively, and 54% yield were achieved. After genetic optimization, the LmrR(V15pAF/M8L) variant with one equivalent of Cu(II)-Phen **95** gave > 99%/93% ee with 82% yield. The ArM was found to tolerate a variety of α,β-unsaturated aldehydes. Thus, the synergistic combination of two abiological moieties in a protein scaffold was demonstrated.

## 4. Rapid Genetic Optimization and Directed Evolution of ArMs

The Nobel Prize in Chemistry awarded in 2018 highlighted directed evolution as one of the most powerful tools to improve the properties of proteins [114]. This technique has been applied to various enzymes to significantly improve their catalytic activity and selectivity. Even a minuscule non-intrinsic reactivity can be greatly enhanced for the catalysis of unnatural reactions through the directed evolution of natural enzymes [115]. However, the directed evolution of ArMs is in the early stages of the development. Upon construction of ArMs, purification of the host protein is conventionally required prior to the introduction of an abiotic cofactor into the host protein, which limits the throughput of the screening process. For the directed evolution of ArMs, maintaining a link between the catalytic activity and the introduced mutation in the host protein is indispensable and needs to be addressed. To this end, Ward and coworkers established a methodology for the construction of an ArM that catalyzed olefin metathesis in the *E. coli* periplasm. This enables rapid screening of catalytic activities of ArM variants with their genetic memories (a plasmid coding a host protein variant) in a multi-well plate-based assay [116].

**Figure 19:**
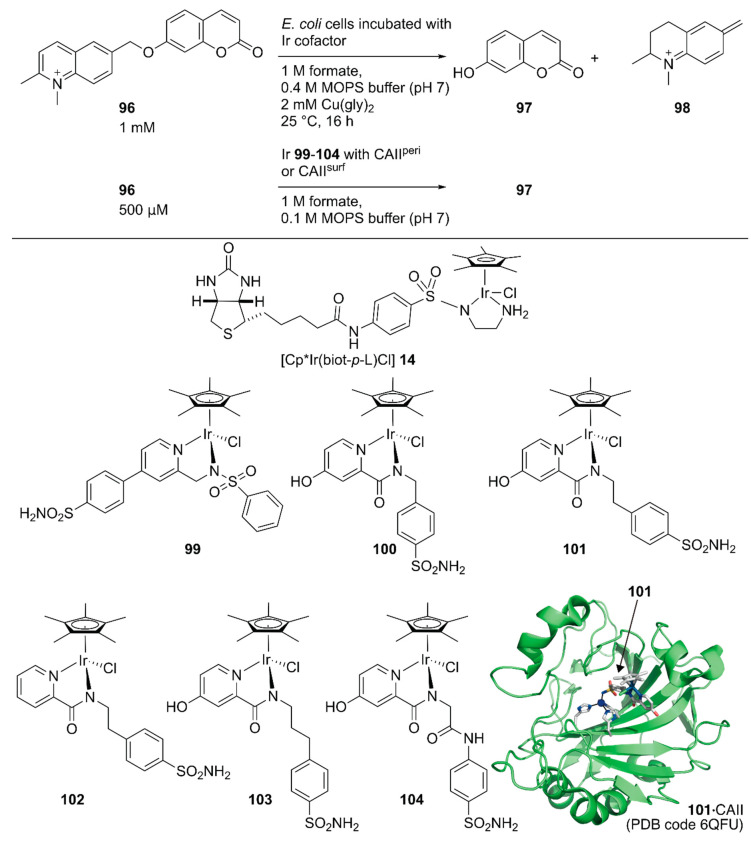
Transfer hydrogenation reaction of a turn-on type fluorescent substrate catalyzed by an ArMs constructed in the periplasm or on the surface of *E. coli.* for high-throughput screening. Adapted from [117,119].

Following the development of this Sav-based artificial metathase that catalyzes olefin metathesis, Ward and coworkers developed a turn-on type fluorescent substrate for the directed evolution of a Sav-based artificial transfer hydrogenase comprising periplasmic Sav and [Cp*Ir(biot-*p*-L)Cl] **14** [117]. The turn-on type fluorescent substrate **96** released fluorescent umbelliferone **97** and **98** upon ArM-catalyzed transfer hydrogenation (Figure 19). This turn on-type fluorescent substrate facilitated the screening of periplasmic Sav derivatives and revealed that the chimeric Sav with an FoldIt Player Design (FPD) peptide (Sav-FPD) provided an improved yield [118]. Directed evolution of Sav-FPD suggested that the Sav-FPD(S112V/K121A) variant was the best protein scaffold. The purified Sav-FPD(S112V/K121A) variant displayed a TON of 819 for **96** within 24 h. The authors also investigated the substrate scope and confirmed that the general trends in the catalytic activity observed in the screening process were similar for other substrates.

Although the strategy to construct ArMs in the periplasmic fraction was successful, passive diffusion of the abiotic cofactor across the *E. coli* outer membrane was deemed necessary for the assembly of ArMs in the periplasm. To circumvent this difficulty in the diffusion of an abiotic cofactor, the construction of ArMs on the *E. coli* surface was proposed as an alternative strategy, as it would allow the maintenance of the linkage between catalytic activity and introduced mutations in a multi-well plate-based rapid screening. Sav was fused with the Lpp-OmpA anchor protein, which consists of the truncated *E. coli* lipoprotein Lpp (residues 1–9) fused to a part of the outer membrane protein OmpA (residues 46–159). Sav displayed on the *E. coli* surface was confirmed by staining the cell surface with a primary mouse-anti Sav antibody and a secondary fluorescent antibody.

**Figure 20:**
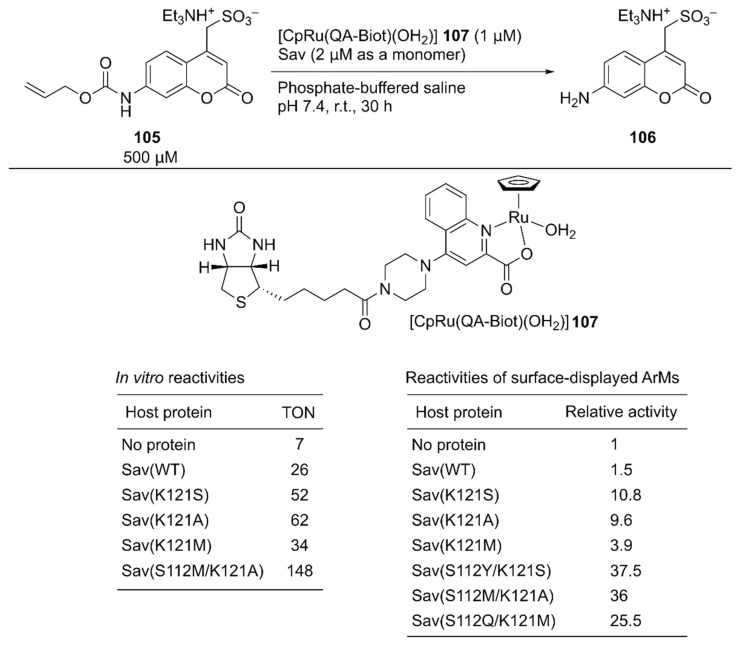
An ArM containing CpRu(2-quinolinecarboxylate) complex **107** was constructed with purified Savs and on the surface-expressed Sav for the allylic dealkylation reaction. Adapted from [120]. See Figure 2 for the crystal structure of Sav.

As a benchmark bio-orthogonal reaction, CpRu(2-quinolinecarboxylate) **107**, which catalyzes allylic dealkylation, was selected because the ruthenium catalyst tolerates a cellular environment [120]. In uncaging the water-soluble pro-fluorogenic substrate **105**, **107** alone gave a TON of 7, whereas the ArM **107**·Sav(WT) afforded a TON of 26 (Figure 20). Having identified the reaction conditions *in vitro*, saturation mutagenesis was performed at the K121 residue, which is the putative amino acid residue near the ruthenium center, with the surface-displayed Sav. Screening revealed that Sav(K121S), Sav(K121A), and Sav(K121M) had improved catalytic activities relative to those of Sav(WT). A second-round saturation mutagenesis at position S112 was performed with Sav(K121S), Sav(K121A), and Sav(K121M) as templates. The highest activity with the surface-displayed system was obtained with Sav(S112Y/K121S) (37.5-fold versus Sav(WT)). It was also confirmed that the purified Sav variants showed significant enhancement of the catalytic activity, as observed in the surface-displayed system. The order of activities of the purified Sav single variants was slightly changed, and the purified Sav(S112M/K121A) displayed the highest activity. The TON improved from 26 with Sav(WT) to 148 with Sav(S112M/K121A) (Figure 20).

Instead of the *E. coli* cell surface, Gademann and coworkers functionalized the surface of other types of cells with an ArM [121]. The unicellular eukaryote *Chlamydomonas reinhardtii* was covalently modified with biotin by a coupling reaction between the amino groups on the cell wall and a succinimidyl ester bearing a biotin moiety. In parallel, Ru cofactor **107** was incorporated into the Sav(S112Y/K121R) mutant to produce an artificial deallylase (Figure 20). The artificial deallylase was then treated with the biotinylated cell to assemble the **107**·Sav(S112Y/K121R)-functionalized cell. The obtained cell could catalyze the cleavage of the *O*-allyl carbamate group of pro-coumarin **105**. Interestingly, the ArM-functionalized cells were still viable and showed negative phototaxis, which allowed the three-dimensional localization of the catalysts by external stimuli.

Ward and coworkers also evaluated human carbonic anhydrase II (CAII) as a scaffold for whole-cell transfer hydrogenation in and on *E. coli* [119]. For in cell compartmentalization, the *N*-terminus of CAII was fused to the signal peptide of the outer membrane protein A (OmpA) to ensure its secretion to the periplasm of *E. coli.* For the surface display, CAII was anchored in the outer membrane by fusing to a truncated *E. coli* lipoprotein Lpp, as described above for the Sav system. The performance of the CAII(WT)-based ArMs were evaluated, both in the *E. coli* periplasm and on its outer membrane (CAII^peri^ and CAII^surf^, respectively) by combining them with six Ir cofactors, **99**–**104** (Figure 19). The expressions of functional CAII^peri^ and CAII^surf^ were confirmed by a fluorescent probe of CAII. The turn-on type fluorescent substrate **96** facilitated the screening of the ArMs’ activities. Cofactors **100**–**104** were markedly superior to **99**, both in vitro and in whole-cell systems, with TONs of up to 93 and 85 for CAII^peri^ and CAII^surf^ possessing cofactor **101**, respectively. Although the expression level of CAII^surf^ was lower than that of CAII^peri^, the amount of bound Ir cofactors and catalytic activities in the surface display was comparable to those of the periplasmic compartmentalization. This suggested more efficient binding of cofactors to proteins in the surface display than the periplasmic compartmentalization, which would be an advantage when using cofactors with low cell permeabilities.

**Figure 21:**
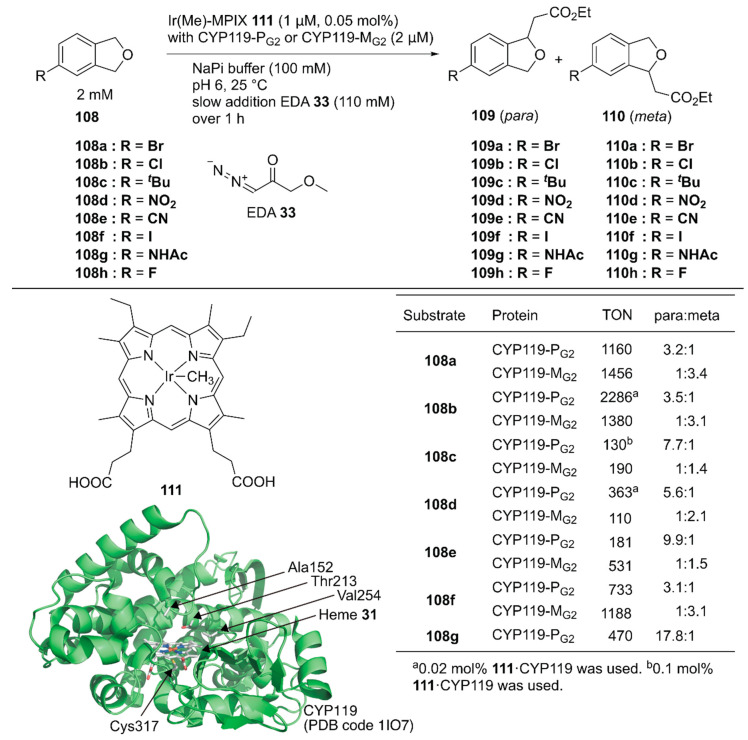
Carbene insertion reaction to phthalane derivatives catalyzed by CYP119 variants reconstituted with the Ir(Me)-MPIX complex **111**. Adapted from [122].

Hartwig and coworkers reported site-selective functionalization of (sp^3^) C–H bonds in phthalan derivatives **108** catalyzed by ArMs based on P450 (CYP119) mutants reconstituted with Ir(Me)-mesoporphyrin IX (MPIX) cofactor **111** (Figure 21) [122]. They previously reported highly efficient carbene insertion into C–H bonds by using this ArM in combination with directed evolution [123]. Phthalan derivatives have two sites of (sp^3^) C–H bonds with similar steric and electronic environments, which are difficult to modify selectively. Indeed, in the reaction between **108a** and ethyl 2-diazoacetate (EDA) **33**, Ir(Me)-MPIX cofactor **111** alone afforded a mixture of products resulting from single and double insertion of the carbene into the (sp^3^) C–H bonds. The products from single carbene insertion at the *meta* **109a** and *para* **110a** positions relative to the bromine atom were observed in a 1:1 ratio. In contrast, the double mutant CYP119(T213G/C317G) reconstituted with **111** exclusively promoted single carbene insertion with negligible *para*/*meta* selectivity. Directed evolution of CYP119(T213G/C317G) led to CYP119-P_G1_ (CYP119(C317G/T213G/V254L/A152F)), which preferentially formed product **109a** over **110a** with a 2.8:1 *para*:*meta* ratio and a TON of 720. In contrast, the other variant, CYP119-M_G1_ (CYP119(C317G/T213G/V254A/A152L)), preferentially formed product **110a** over **109a** with a 1:2.7 *para*:*meta* ratio and a TON of 690. CYP119-P_G1_ and CYP119-M_G1_ were further optimized by using error-prone polymerase chain reaction (PCR) and obtained CYP119-P_G2_ and CYP119-M_G2_, respectively. CYP119-P_G2_ and CYP119-M_G2_ exhibited *para*/*meta* selectivity for a broad range of phthalan derivatives (Figure 21). CYP119-P_G2_ could also catalyze carbene insertion into amide-substituted phthalan derivatives such as **108g**, containing a reactive N–H group, with 17.8:1 selectivity for **109g** over **110g**. It was also found that an enantiomeric ratio of 97:3 was obtained for the insertion of EDA into 4-fluorophthalan **108h**. Thus, directed evolution of hemoproteins reconstituted with the non-canonical heme analog achieved regioselectivities for abiotic reactions that would be hard to attain with small-molecule catalysts.

**Figure 22:**
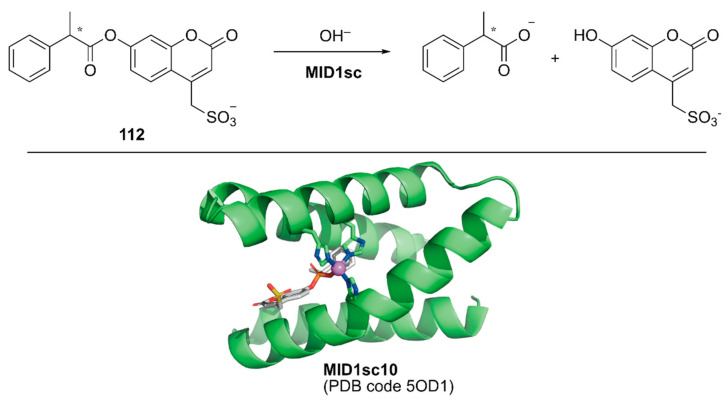
Hydrolysis reaction catalyzed by MID1sc and the crystal structure of MIDsc10 (PDB code: 5OD1). Adapted from [124]. The peptide scaffold is depicted as a green cartoon. Carbon, nitrogen, oxygen, phosphorus, and sulfur atoms are shown in green, blue, red, orange, and yellow, respectively. The Zn ion is shown as a purple sphere. A phosphonate transition state analog is shown as a gray stick structure.

Directed evolution enables the development of an ArM with prominent activity from even simple peptides. Hilvert and coworkers conducted directed evolution of a Zn-binding peptide to obtain a de novo enzyme with catalytic activity comparable to that of natural enzymes [124]. From a monomeric forty-six amino acid-long helix–turn–helix fragment, a homodimeric peptide with two Zn(II)His_3_ sites (hereafter MID1) was computationally designed [125]. Prior to directed evolution, MID1 was redesigned to MID1sc, in which adjacent N and C termini of the dimer subunits were connected via a Gly–Ser–Gly linker and one of the two zinc sites farthest from the linker was removed by replacing the metal-binding residues with non-coordinating amino acids. MID1sc was optimized for the hydrolysis of ester **112** (Figure 22) by nine rounds of evolution, including cassette mutagenesis, DNA shuffling, and random mutagenesis, and elimination of the competitive zinc-binding sites to give MID1sc10. Interestingly, during the evolution, the Zn-binding site was shifted from His39, His61, and His65 in the original MID1sc to His35, His61, and His65. The hydrolysis reaction performed by MID1sc10 had *k*_cat_ = 1.64 ± 0.04 s^−1^, *k*_cat_/*K*_M_ = 980,000 ± 110,000 M^−1^ s^−1^, and 990-fold kinetic preference for the (*S*)-configured ester. Optimization of the catalytic activity from a simple peptide scaffold drastically converted the coordination structure of the metal complex inside the peptide, illustrating a plausible enzyme evolutionary pathway from a peptide and providing a new insight for enzyme design and engineering.

## 5. Drug Applications

A living system consists of myriad biochemical reaction networks. In an engineering spirit, chemists and biologists have redesigned these biochemical reaction networks using either isolated enzymes [126] or transgene-encoded proteins in cells [127,128]. In the former case, compartmentalization approaches including ArMs enable the integration of synthetic catalysts into enzymatic cascades, as discussed in the above section [24,34,40,99,100,101]. The next challenge in the field is achieving unnatural reactions at an intracellular level. In chemical biology, various intracellular stoichiometric chemical transformations, such as the detection of biomolecules by fluorescent probes, have been developed. In contrast, catalytic chemical transformations (intracellular catalysis) are still in the early stages of development.

**Figure 23:**
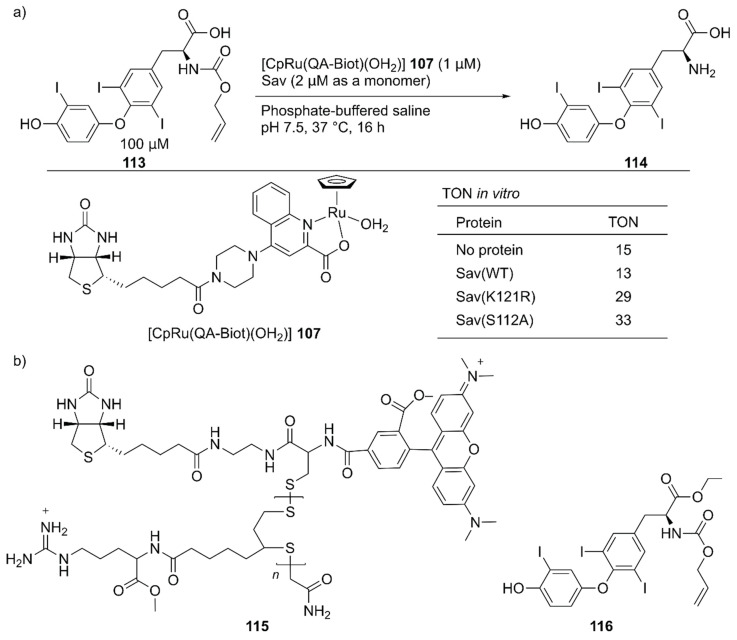
(**a**) Cleavage of the *O*-allyl carbamate of caged T_3_ hormone AT_3_ **113** catalyzed by artificial deallylase **107**·Sav and selected results of its in vitro activity. Adapted from [136]. See Figure 2 for the crystal structure of Sav. (**b**) A cell-penetrating ArM **107**–**115**·Sav and the double-caged T_3_ hormone AM-AT_3_ **116** for upregulation of gene expression.

Several examples of intracellular organometallic catalysis within *E. coli* or mammalian cells have been reported [129,130,131,132,133,134,135]; however, most of these catalytic transformations produced bio-orthogonal outputs. To realize productive cooperation between cellular function and an unnatural reaction, the group of Ward collaborated with Matile et al. to make ArMs cell permeable and with Fussenegger et al. to design a cellular function that could be connected to abiotic catalysis [136]. Upregulation of protein expression was selected as a target cellular function modulated by an ArM. HEK-293T cells were implemented with a thyroid hormone (T_3_ **114**) responsive gene switch. In these designer HEK-293T cells, T_3_ **114** induced the expression of secreted nanoluc (sec-nluc), a bioluminescence reporter. Based on the Meggers’ ruthenium catalyst for the uncaging of an *O*-allyl carbamate [133], the artificial deallylase **107**·Sav was utilized to produce T_3_ **114** from caged AT_3_ **113** in situ. Through the in vitro genetic optimization of artificial deallylase **107**·Sav, it was found that the Sav(S112A) variant showed the highest activity for uncaging **113** (Figure 23). Because AT_3_ **113** needs to diffuse into the cell, its carboxylate group was esterified to obtain AM-AT_3_ **116** for more favorable passive diffusion. The acetoxymethyl group of AM-AT_3_ **116** was readily hydrolyzed by endogenous esterases in the cell to give AT_3_ **113**. For efficient cellular uptake, the artificial deallylase **107**·Sav(S112A) was equipped with the cell-penetrating poly(disulfide) (CPD) **115**. The tetrameric feature of Sav enabled the co-assembly of the biotinylated abiotic cofactor **107** and biotinylated CPD **115**. The fluorescent carboxytetramethylrhodamine (TAMRA) moiety contained in the CPD allowed confirmation of cellular uptake and localization of the artificial deallylase **107**–**115**·Sav(S112A). Finally, the designer HEK-293T cells transfected with the T_3_-responsive gene switch were treated with **107**–**115**·Sav(S112A). Cellular uptake of **107**–**115**·Sav(S112A) was confirmed by an increase in fluorescence derived from the TAMRA moiety. Increased expression of sec-nluc was observed when **107**–**115**·Sav(S112A) was used, indicating that an enzymatic cascade comprising endogenous esterase and artificial deallylase proceeded to convert **116** into **114**. In contrast, neither **107** alone nor **107**·Sav(S112A) could turn on the T_3_-responsive gene switch. Subsequently, it was also demonstrated that the artificial deallylase **107**·Sav could be introduced into HeLa cells by using different types of cell-penetrating cargo and could successfully show catalytic activity [137,138].

**Figure 24:**
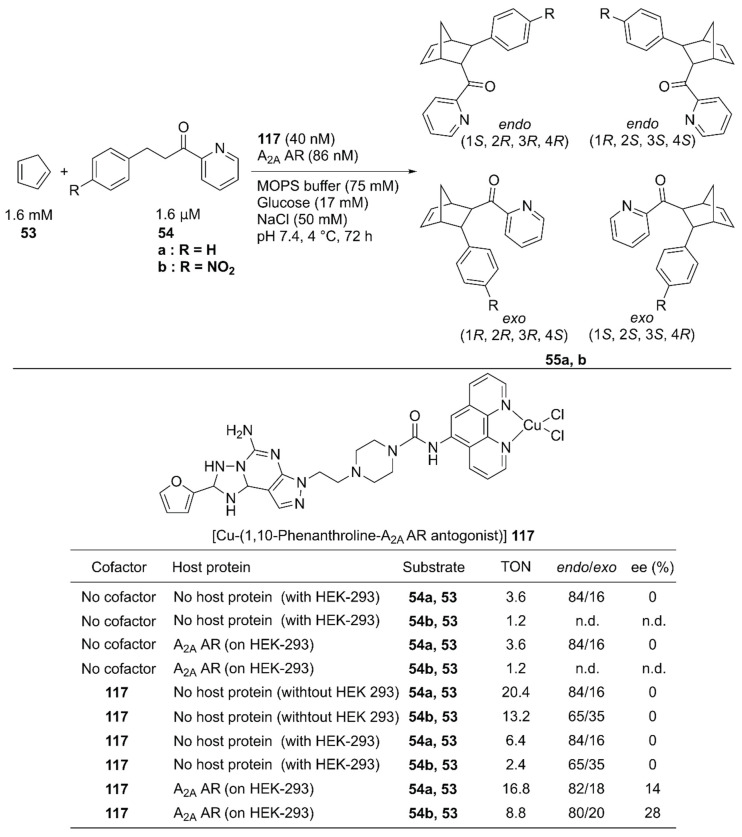
Cell-surface Diels–Alder reaction catalyzed by **117**·A_2A_. Adapted from [78].

In vivo construction of an ArM by using a protein expressed in a specific cell line potentially contributes to the methodology of in situ drug synthesis. With this therapeutic application in mind, Mahy and coworkers utilized the wild-type human A_2A_ adenosine receptor (AR) at the surface of HEK-293 cells as a host protein of the Cu-Phen complex **117** and used it to catalyze the Diels–Alder reaction of **53** and **54** (Figure 24) [78]. A Phen ligand was attached to an antagonist of the A_2A_ AR. Because the antagonist was utilized as an anchor, no cellular response was induced upon binding of **117** (*K*_d_ = 3.8 nM). **117** alone catalyzed the reaction of **53** and **54a**, giving a TON of 20.4 with 84/16 *endo*/*exo* ratio, and no enantiomeric excess. A background reaction (TON = 3.6 for **53** and **54a**) was also observed. This was attributed to either template effects in the different hydrophobic cavities of proteins on a cell surface or some leached transition metals possessing Lewis acid properties. In the presence of HEK-293 cells, **117** alone afforded a TON of 6.4 with no enantiomeric excess for **55a**. In contrast, **117** combined with A_2A_ AR expressed on HEK-293 cells gave a TON of 16.8 with 28% ee, indicating that the formation of the ArM is beneficial for the catalytic activity in the presence of the cell. The *endo*/*exo* ratio of products from **53** and **54a** was also affected, as the *endo* isomer was further favored when the ArM was formed on the cell surface.

**Figure 25:**
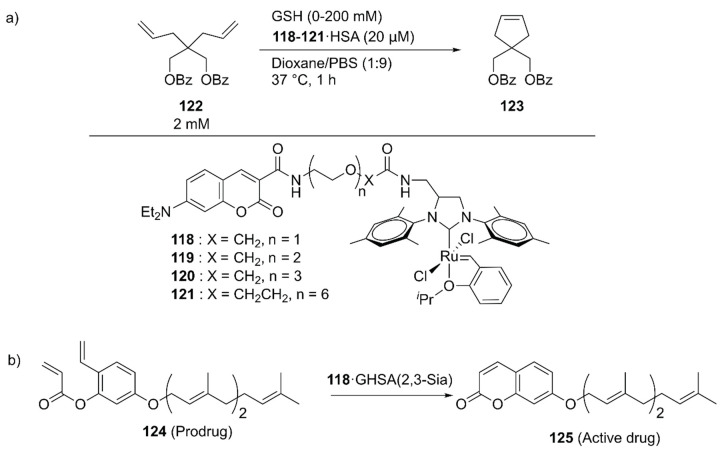
(**a**) RCM reaction catalyzed by GH-type Ru catalysts combined with HSA. (**b**) Prodrug activation by **118**·GHSA(2,3-Sia) generating umbelliprenin **125**. Adapted from [139].

With further pharmaceutical applications of ArMs in mind, Tanaka and coworkers linked the GH catalysts **118**–**121** to human serum albumin (HSA) via supramolecular interaction between the coumarin moiety of the catalyst and drug site I in HSA (Figure 25) [139]. The catalytic activities of the obtained ArMs toward ring-closing metathesis (RCM) decreased as the linker length increased from **118** to **121**, suggesting the importance of residual space in the hydrophobic pocket to maintain the reactivity. Next, the catalytic activity of **118**·HSA for the RCM reaction of **122** to produce **123** was evaluated in the presence of glutathione (GSH) to check the biocompatibility. Because the negatively charged surface of HSA prevented the binding of negatively charged GSH to **118**, **118**·HSA retained its catalytic activity up to 20 mM GSH. The researchers further investigated the targetability of the obtained ArMs toward cancer cells by attaching α (2,3)-linked sialic acid-terminated *N*-glycan to the surface of HSA, yielding a glycosylated ArM (**118**·GHSA(2,3-Sia)) (Figure 25). As expected, **118**·GHSA(2,3-Sia) accumulated preferentially on the SW620 colon cancer cells that overexpress galactin-8, a receptor of α(2,3)-linked sialic acid. Prodrug activation, in which prodrug **124** was converted into umbelliprenin **125** via RCM by **118**·GHSA(2,3-Sia), was also tested. SW620 cell growth was remarkably inhibited in the presence of prodrug **124** and **118**·GHSA(2,3-Sia), illustrating the potential application of **118**·GHSA(2,3-Sia) for prodrug therapy.

**Figure 26:**
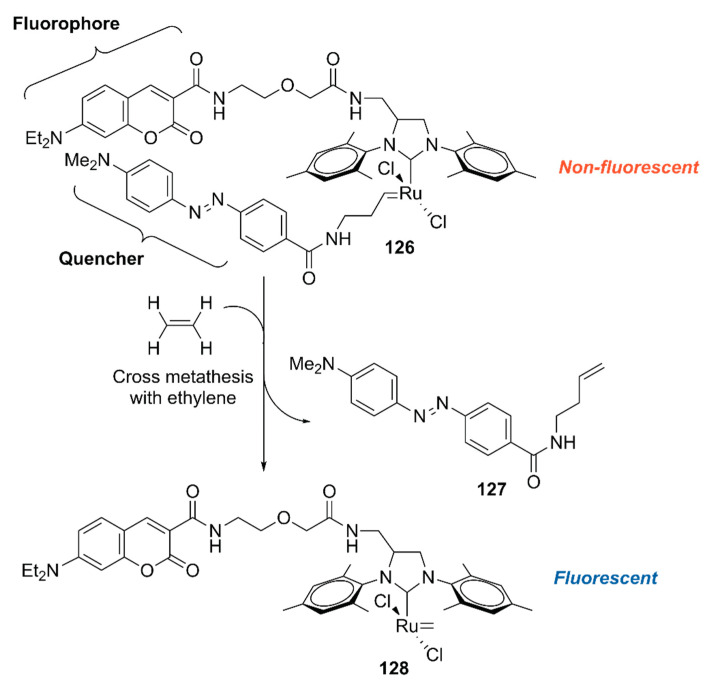
Ethylene-dependent fluorescence switching by a ruthenium complex [140].

To explore the application of ArMs as diagnostic tools, **118**·HSA was modified with **127** to give an ethylene probe **126**·HSA (Figure 26) [140]. In the presence of ethylene, cross-metathesis of **126** with ethylene occurs to dissociate the fluorescent quencher **127**, recovering the fluorescence of the coumarin moiety. As an ethylene probe, ArMs have several advantages over other molecular probes; for example, there is no need for an organic solvent to dissolve the probe and the protein scaffold can protect the metal catalyst from biological thiols [139]. In this study, **126**·HSA was utilized for the spatial and temporal detection of ethylene in some fruits and a plant *Arabidopsis thaliana*, which has effectively paved the way for ArM-based biosensors.

**Figure 27:**
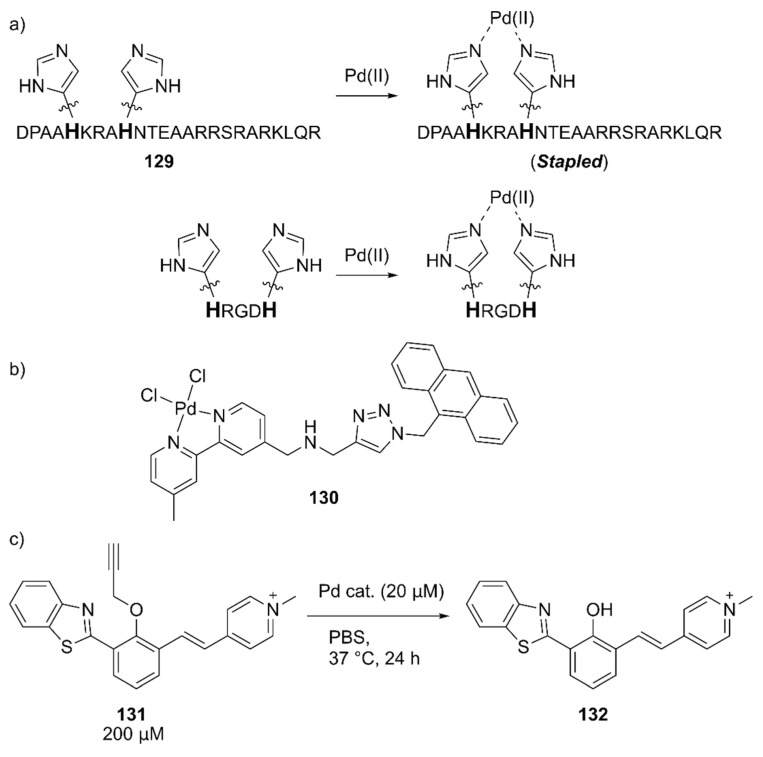
(**a**) Pd(II) coordination to **129** and HRGDH peptides. Adapted from [141]. (**b**) Structure of **130**. (**c**) Pd-catalyzed in vitro uncaging reaction of **131**.

Mascareñas and coworkers evaluated the intracellular reactions within mammalian cells by using Pd(II)-coordinated peptides [141]. These authors previously discovered that introducing two histidine residues in the *i* and *i* + 4 positions of the basic region of the GCN4 bZIP transcription factor **129** triggered an efficient membrane translocation into the mammalian cells upon addition of a Pd(II) source by stapling the peptide (Figure 27) [142]. They first confirmed the importance of the stapled peptide on the cell internalization of Pd(II) using tetramethylrhodamine (TMR)-labeled **129** and the fluorescent **130** complex (Figure 27). The importance of arginine residues was also noted based on the observation that the lack of one of these residues significantly reduced the cell internalization. Next, the in vitro reactivity of Pd(II)-complexed **129** toward the uncaging of **131** to form fluorescent **132** was assessed (Figure 27). It was found that dichloro(1,5-cyclooctadiene)palladium(II), (hereafter PdCl_2_(cod), cod = 1,5-cyclooctadiene) gave the best result with a TON of > 5 when combined with **129**. The intracellular reactions were finally performed by the sequential treatment of HeLa cells with **131** and PdCl_2_(cod)·**129**, which resulted in successful uncaging of **131** in the cell. These researchers further discovered that the shorter peptide HRGDH, in which RGD is an integrin-targeting motif, could also perform cell internalization and the cellular uncaging reaction upon addition of PdCl_2_(cod). The reactivity of PdCl_2_(cod)-complexed HRGDH peptide was high in A549 and HeLa cells and low in the MCF-7 breast cancer cell line, reflecting the integrin expression level. This work demonstrated for the first time that synthetic peptides equipped with a metal complex can promote abiotic reactions inside living mammalian cells.

## 6. Conclusions and Perspectives

In this review, the recent trends in ArM research are presented. Even though only a few years have passed since the last comprehensive review of ArMs by Ward et al., several dozen articles have been published [7]. This review includes ArM research reports focused on the construction of new ArMs from the perspective of catalyst development, their directed evolution, and the use of non-natural amino acids as a construction methodology. For directed evolution, it is necessary to link the catalytic activities of ArMs with the mutations introduced into the host protein, which has been successfully achieved by constructing ArMs in or on *E. coli*. [116,120]. To be at par with the directed evolution of natural enzymes [4,5,6], however, that of ArMs needs to be further developed.

Upon construction of an ArM, the removal of any excess abiotic cofactor and/or non-specifically bound abiotic cofactor has been a problem except for examples relying on highly selective modification systems such as the biotin–avidin technology. In contrast, for non-natural amino acid-based ArMs, the abiotic cofactor is precisely incorporated into the protein upon its expression. If the repertoire of non-natural amino acids is greatly expanded, this has the potential to become one of the main methods for constructing ArMs in the future.

Applications of ArMs have steadily increased in the past few years. Starting with the artificial-natural enzymatic cascade reported by Ward et al. in 2013 [24], reaction networks using different classes of catalysts, such as ArMs and natural enzymes, have been designed. These reaction networks only work when an ArM is used, but not with their cofactors alone. By extending this concept, ArMs have been used in and on mammalian cells for pharmaceutical applications [78,136,139,140,141]. Thus, we believe that ArMs have great potential as catalysts for organic synthesis and as chemical biology tools for pharmaceutical applications.

## Figures and Tables

**Table 1:** Selected results of transfer hydrogenation of imines catalyzed by Sav-based artificial transfer hydrogenases [35,36,37,38].

Entry	Cofactor (cof)	µM	Protein	Substrate	pH	T (°C)	Time (h)	ee %	TON	Ref
1	(*S*)-**12**	190	No protein	**8**	7.8	30	18	3*R*	101	[35]
2	(*S*)-**12**	190	Sav (WT)	**8**	7.8	30	18	7*R*	43	[35]
3	(*S*)-**12**	190	Sav(K121A)	**8**	7.8	30	18	13*R*	43	[35]
4	(*R*)-**13**	190	No protein	**8**	7.8	30	18	65*R*	110	[35]
5	(*R*)-**13**	190	Sav (WT)	**8**	7.8	30	18	43*R*	13	[35]
6	(*R*)-**13**	190	Sav (S112M) (cof:Sav = 1.5:4.0)	**8**	7.8	30	18	68*R*	30	[35]
7	(*R*)-**13**	190	Sav (S112M) (cof:Sav = 2.5:4.0)	**8**	7.8	30	18	83*R*	33	[35]
8	**14**	50	No protein	**8**	7.0	37	48	rac	65	[36]
9	**14**	50	Sav (S112A)	**8**	7.0	37	48	75*R*	142	[36]
10	**14**	50	Sav (S112K)	**8**	7.0	37	48	41*S*	100	[36]
11	**14**	50	Sav (S112A/K121A)	**8**	7.0	37	48	59*R*	358	[36]
12	**14**	0.42	Sav (S112A) @ Ferritin	**8**	7.0	37	48	46*S*	74	[36]
13	**14**	0.40	Sav (S112K) @ Ferritin	**8**	7.0	37	48	47*S*	46	[36]
14	**14**	0.28	Sav (S112A/K121A) @ Ferritin	**8**	7.0	37	48	44*S*	117	[36]
15	**14**	10	No protein	**15**	7.5	37	16	n.d.	0	[37]
16	**14**	10	Sav	**15**	7.5	37	16	76*R*	22	[37]
17	**14**	10	Sav_HP46-52 Inserted seq: LSDEDFKAVFGMTRSAFANLPLWKQQHLKKEKGLF	**15**	7.5	37	16	80*R*	162	[37]
18	**14**	10	Sav_FPD46-52 Inserted seq: SPLSEALTKANSPAEAYKASRGAG	**15**	7.5	37	16	82*R*	158	[37]
19	**14**	25	No protein	**8**	7.0	25	24	rac	72	[38]
20	**14**	25	Sav	**8**	7.0	25	24	30*R*	88	[38]
21	**14**	25	scdSav (112S^A^/121K^A^/112S^B^/121K^B^)	**8**	7.0	25	24	73*R*	176	[38]
22	**14**	25	scdSav (112S^A^/121A^A^/112R^B^/121K^B^)	**8**	7.0	25	24	96*R*	400	[38]
23	**14**	5	monovalent scdSav (112S^A^/121A^A^/112R^B^/121K^B^)	**8**	7.0	50	48	90*R*	17700	[38]
24	**14**	50	scdSav (112S^A^/121K^A^/112A^B^/121A^B^)	**15**	7.0	37	48	96*R*	1976	[38]
25	**14**	50	monovalent scdSav (112S^A^/121A^A^/112R^B^/121K^B^)	**17**	7.0	50	48	91*R*	195	[38]

**Table 2:** Selected results of oxidation reactions catalyzed by acylated protein-bearing metal ions in organic solvents [47].

Entry	Metal Source	Host Protein	Modification	[Metal] (µM)	Substrate	Product	tBuOOH (mM)	T (°C)	Time (h)	TON	ee%
1	K_2_OsO_2_	Lysozyme	HA	30	**23**	**24**	110	0	168	289	98*S*
2	K_2_OsO_2_	Lysozyme	PA	30	**23**	**24**	110	0	168	85	94*S*
3	K_2_OsO_2_	Lysozyme	AA	30	**23**	**24**	110	0	168	103	55*S*
4	K_2_OsO_2_	BSA	AA	30	**23**	**24**	110	0	168	275	73*S*
5	RuCl_3_	Lysozyme	No modification	60	**23**	**25**	220	20	72	361	10*R*
6	RuCl_3_	Lysozyme	HA	60	**23**	**25**	220	20	72	449	56*R*
7	RuCl_3_	Lysozyme	PA	60	**23**	**25**	220	20	72	561	74*R*
8	RuCl_3_	Lysozyme	AA	60	**23**	**25**	220	20	72	749	63*R*
9	RuCl_3_	BSA	HA	60	**23**	**25**	220	20	72	452	88*R*
10	RuCl_3_	BSA	HA	180	**23**	**25**	220	40	72	2613	82*R*

**Table 3:** Selected results of cyclopropanation catalyzed by ArMs containing iron porphyrinoids as their cofactor [55,59,62].

Entry	Cof	Host Protein	[Cof] (µM)	Substrate	pH	T (°C)	Time (h)	TON	de for (*E*)	ee% (1*R*,2*R*)	Ref
1	**31**	No protein	4.3	**23**, **33**	8.0	25	60	23	62	n.d.	[55]
2	**31**	Mb	4.3	**23**, **33**	8.0	25	60	49	85	n.d.	[55]
3	**32**	No protein	4.3	**23**, **33**	8.0	25	6	9	53	n.d.	[55]
4	**32**	Mb	4.3	**23**, **33**	8.0	25	6	133	> 99	n.d.	[55]
5	**31**	No protein	10	**33**, **35**	8.0	4	18	51	>70	–	[59]
6	**31**	LmrR	10	**33**, **35**	8.0	4	18	247	>70	17	[59]
7	**31**	LmrR (F93A)	10	**33**, **35**	8.0	4	18	232	>70	11	[59]
8	**31**	LmrR (D110A)	10	**33**, **35**	8.0	4	18	375	>70	24	[59]
9	**31**	LmrR (W96A)	10	**33**, **35**	8.0	4	18	276	>70	<5	[59]
10	**31**	LmrR (V15A)	10	**33**, **35**	8.0	4	18	15	>70	17	[59]
11	**31**	LmrR (M8A)	10	**33**, **35**	8.0	4	18	359	>70	44	[59]
12	**31**	LmrR (M8A)	10	**33**, **35**	7.0	4	18	449	>70	51	[59]
13	**31**	No peptide	20	**33**, **37**	7.0	–	1	60	70	0	[62]
14	**31**	LILHLFL	20	**33**, **37**	7.0	–	1	208	68	–40	[62]
15	**31**	LHLH (l-NMe) FL	20	**33**, **37**	7.0	–	1	64	80	0	[62]
16	**31**	(d)-LILHLFL	20	**33**, **37**	7.0	–	1	200	–	40	[62]

**Table 4:** Selected results of Diels–Alder reactions catalyzed by artificial copper enzymes [79,83].

Entry	Cof	Protein	[Cof] (µM)	[53] (mM)	[54a] (mM)	Yield (%)	Time (h)	TON	*endo*/*exo*	ee (%) for 1*S*,2*R*,3*R*,4*R*	Ref
1	Cu-Phen **51**	A3-A3′(Y26C)	30	34	1	21	48	7.0	n.d.	22	[79]
2	Cu-Terpy **52**	A3-A3′(Y26C)	30	34	1	16	48	5.3	n.d.	14	[79]
3	Cu-Phen **51**	A3-A3′(F119C)	30	34	1	38	48	12.7	92/8	5	[79]
4	Cu-Terpy **52**	A3-A3′(F119C)	30	34	1	15	48	5.0	93/7	52	[79]
5	Cu^2+^ ion	No protein	100	33	1	82	72	8.2	92:8	< 5	[83]
6	Cu^2+^ ion	NB4	100	33	1	16	72	1.6	89:11	–6	[83]
7	Cu^2+^ ion	NB4-Pyr **56**	100	33	1	56	72	5.6	95:5	69	[83]
8	Cu^2+^ ion	NB4-Pyr **56**	100	33	0.25	94	72	2.4	96:4	78	[83]
